# Supplementary material for: Protocol for process evaluation of ARTEMIS cluster randomised controlled trial: an intervention for management of depression and suicide among adolescents living in slums in India
Source: BMJ Open. 2024 May 20;14(5):e081844. doi: 10.1136/bmjopen-2023-081844 (PMC11110603; doi:10.1136/bmjopen-2023-081844)
Supplement: Author's manuscript [file bmjopen-2023-081844.draft_revisions.pdf]

BMJ Open

BMJ Open is committed to open peer review. As part of this commitment we make the peer review history of every article we publish publicly available.

When an article is published we post the peer reviewers' comments and the authors' responses online. We also post the versions of the paper that were used during peer review. These are the versions that the peer review comments apply to.

The versions of the paper that follow are the versions that were submitted during the peer review process. They are not the versions of record or the final published versions. They should not be cited or distributed as the published version of this manuscript.

BMJ Open is an open access journal and the full, final, typeset and author-corrected version of record of the manuscript is available on our site with no access controls, subscription charges or pay-per-view fees (http://bmjopen.bmj.com).

If you have any questions on BMJ Open's open peer review process please email info.bmjopen@bmj.com

BMJ Open

Protocol for process evaluation of ARTEMIS cluster randomized controlled trial: an intervention for management of depression and suicide among adolescents living in slums in India

Journal:	*BMJ Open*
Manuscript ID	bmjopen-2023-081844
Article Type:	Protocol
Date Submitted by the Author:	08-Nov-2023

Complete List of Authors:	Mukherjee, Ankita; The George Institute for Global Health India,<br>Yatirajula, Sandhya Kanaka; The George Institute for Global Health India, Research<br>Kallakuri, Sudha; The George Institute for Global Health<br>Paslawar, Srilatha; The George Institute for Global Health India, Research and Development<br>Lempp, Heidi; King's College London, Inflammation Biology<br>Raman, Usha; University of Hyderabad, Hyderabad, India, Department of Communication,<br>Essue, Beverley; University of Toronto, Institute of Health Policy, Management and Evaluation<br>Sagar, Rajesh; All India Institute of Medical Sciences, Department of Psychiatry<br>Singh, Renu; Young Lives, India<br>Peiris, David; The George Institute for Global Health; University of New South Wales<br>Norton, Robyn; The George Institute for Global Health; Imperial College London<br>Thornicroft, Graham; Institute of Psychiatry, Psychology and Neuroscience, King's College London, Centre for Global Mental Health and Centre for Implementation Science<br>Maulik, Pallab; The George Institute for Global Health; University of New South Wales Medicine & Health

Keywords:	Adolescents < Adolescent, Protocols & guidelines < HEALTH SERVICES ADMINISTRATION & MANAGEMENT, Implementation Science, MENTAL HEALTH, Feasibility Studies, Clinical Trial

SCHOLARONE™
Manuscripts

Protocol for process evaluation of ARTEMIS cluster randomized controlled trial: an intervention for management of depression and suicide among adolescents living in slums in India

Authors

1. Ankita Mukherjee (AM), The George Institute for Global Health, New Delhi, India, Email - AMukherjee@georgeinstitute.org.in>
2. Sandhya Kanaka Yatirajula (SKY), Corresponding author), The George Institute for Global Health, New Delhi, India, Email – syatirajula@georgeinstitute.org.in
3. Sudha Kallakuri (SK), The George Institute for Global Health, Hyderabad, India, Email - skallakuri1@georgeinstitute.org.in
4. Srilatha Paslawar (SP), The George Institute for Global Health, Hyderabad, India, Email - SPaslawar@georgeinstitute.org.in>
5. Heidi Lempp (HL), Centre for Rheumatic Diseases, Department of Inflammation Biology, School of Immunology and Microbial Sciences, Faculty of Life Sciences and Medicine, King's College London, UK, Email - heidi.lempp@kcl.ac.uk
6. Usha Raman (UR), Department of Communication, University of Hyderabad, Hyderabad, India, Email - usharaman@uohyd.ac.in
7. Beverley M. Essue (BE), Institute for Health Policy, Management and Evaluation, University of Toronto, Toronto, Canada, Email - beverley.essue@utoronto.ca
8. Rajesh Sagar (RS), Department of Psychiatry, All India Institute of Medical Sciences, New Delhi, Email - rsagar29@gmail.com
9. Renu Singh (ReS), Young Lives India, New Delhi, Email - renusab@gmail.com
10. David Peiris (DP), The George Institute for Global Health, UNSW Sydney, Sydney Australia, Email - dpeiris@georgeinstitute.org
11. Robyn Norton (RS), The George Institute for Global Health, UNSW Sydney, Sydney, Australia Imperial College, London, Email - rnorton@georgeinstitute.org
12. Graham Thornicroft (GT), Centre for Global Mental Health and Centre for Implementation Science, Institute of Psychiatry, Psychology and Neuroscience, King's College London, UK, Email - graham.thornicroft@kcl.ac.uk
13. Pallab K Maulik (PKM), The George Institute for Global Health, New Delhi, India, University of New South Wales, Sydney, Australia, Department of Brain Sciences, Imperial College London, London, United Kingdom, George Institute for Global Health, Imperial College London, United

For peer review only

For peer review only - http://bmjopen.bmj.com/site/about/guidelines.xhtml

Kingdom, Prasanna School of Public Health, Manipal Academy of Higher Education, Manipal, India, Email - pmaulik@georgeinstitute.org.in

For peer review only

For peer review only - http://bmjopen.bmj.com/site/about/guidelines.xhtml

Abstract

Introduction

There are around 250 million adolescents (10-19 years) in India. The prevalence of mental health related morbidity among adolescents in India is approximately 7.3%, Certain vulnerable sub-populations among adolescent such as those living in slum communities, are particularly at risk due to poor living conditions, financial difficulty, and limited access to support services. There has been limited research to test community-based mental health interventions for adolescents living in slums in India. The Adolescents' Resilience and Treatment nEeds for Mental Health in Indian Slums (ARTEMIS) is a cluster randomized controlled trial (cRCT) of an intervention intends to improve mental health of adolescents living in slum communities in India. It involves an anti-stigma campaign and a mobile device based electronic decision support system for primary care doctors to manage depression, and suicide for high-risk adolescents living in urban slums. The aim of this paper is to describe the process evaluation protocol for ARTEMIS trial.

Methods

Case study method will be used and the data will include a mix of quantitative metrics and qualitative data. The UK Medical Research Council's guidance on evaluating complex interventions, the RE-AIM Framework and the APEASE criteria will inform the design and analysis plan of the process evaluation. Implementation fidelity will also be measured.

Discussion

The process evaluation will provide an understanding of outcomes and causal mechanisms that influenced any change in trial outcomes. It will identify contextual factors, intervention barriers and facilitators and the adaptations required for optimising implementation.

Ethics and Dissemination

Ethics approval was obtained from the Ethics Committee of the George Institute for Global Health India (Project number 17/2020) and from the Research Governance and Integrity Team, Imperial College, London (ICREC Reference number: 22IC7718). The Health Ministry's Screening Committee, Indian Council for Medical Research has also approved to the study (ID 2020-9770).

Trial Registration

The trial is registered with Clinical Trial Registry of India (Reference Number CTRI/2022/02/040307). The George Institute for Global Health, India is the trial sponsor.

For peer review only - http://bmjopen.bmj.com/site/about/guidelines.xhtml

Article Summary:

Strengths and limitations of this study

- This study will use existing implementation science theories and frameworks and qualitative and quantitative data will be triangulated to arrive at a comprehensive understanding of the intervention.
- This study will be guided by the MRC framework to developing and evaluating complex interventions and the case study method will also be used.
- Cases will be purposively selected based taking a maximum variation approach and both intervention and control sites will be selected as cases to the enable comparison, to understand contextual factors and to avoid the Hawthorne effect.
- This study will not include an economic evaluation, which will be conducted separately.

Key Words: process evaluation, protocol, Child and Adolescent Mental Health (CAMH), Low-middle income country (LMIC), cluster Randomized Control Trial (cRCT), depression, self-harm, anti-stigma, mHealth, electronic decision support system (EDSS), urban slum, India

Word count: 3,300

For peer review only

INTRODUCTION

India has about 250 million adolescents aged 10-19 years, comprising nearly one-fifth of India's population [1]. The burden of mental health problems in adolescents is a growing concern globally[2]. In India, the prevalence of psychiatric disorders among adolescents is estimated to be about 7.3% [3]. Self-harm and depressive disorders are the leading cause of death and disability in the age group 15-19 years [4].

Mental health interventions for adolescents are important as 50% of adult mental disorders have an onset before age of 14 years [5]. Marginalized populations such as adolescents living in slum communities, are particularly vulnerable because of additional stressors related to poor living conditions, financial stress, and poor access to support services [6,7].

There has been limited research to test community-based mental health interventions for adolescents living in slums in India. A scoping review of mental health interventions among adolescents in India found that of the 11 interventions included in the review; nine were school-based, one community-based, and one was digital [8]). Most of the school-based programs used a life skills curriculum that resulted in improvements in depressive symptoms and overall mental well-being. The review recommended the need for more interventions for early and out-of-school adolescents to ensure that the most vulnerable adolescents were not missed out. An intervention to build mental health and resilience delivered by community-based peers among highly disadvantaged young women living in urban slums in Dehradun a city in north India, showed sustained improvements in anxiety and depression and attitudes to gender equality among study participants [9]. A randomized controlled trial of a 5-month resilience-based program among rural adolescent girls through government schools in the state of Bihar, India, delivered by local women showed that girls receiving the intervention (vs. controls) had better emotional resilience, self-efficacy, social-emotional assets, psychological wellbeing, and social wellbeing [10].(Another school-based pilot study of POD (**P**roblems, **O**ptions, **D**o it!) Adventures (a smart phone based blended problem-solving game-based intervention for adolescents with or at risk of anxiety, depression and conduct difficulties) was helpful in managing their problems and stress and improving the mental health of 13 to 19 year olds enrolled in secondary schools in the Indian state of Goa [11]. The Adolescents' Resilience and Treatment nEeds for Mental Health in Indian Slums (ARTEMIS) cluster randomized control trial (cRCT) is testing a community-based intervention to improve mental health outcomes for adolescents living in urban slum clusters in India. For this study, a slum cluster is defined as slums within wards or geographical areas identified as slums/resettlement colonies. A ward is a local authority area, typically used for electoral purposes. In certain cities of India, such as Mumbai and New Delhi, a ward is an administrative unit of the city region; a city area is divided into zones, which in turn contains numerous wards.

For peer review only - http://bmjopen.bmj.com/site/about/guidelines.xhtml

ARTEMIS is a community-based cluster randomised control trial that aims to reduce depression and risk of suicide among adolescents living in slums. The intervention will use a mental health stigma reduction campaign with adolescents and parents of the study cohort to improve attitudes towards mental health and improve help seeking. A technology-enabled strategy will be employed for screening, clinical diagnosis, and management of mental health problems (depression, other significant emotional or medically unexplained complaints and suicide risk) among adolescents by primary care doctors and community based non physician health workers (NPHWs) (described in detail below).

This paper presents the protocol for process evaluation of the ARTEMIS cRCT. Process evaluations provide critical inputs in understanding how interventions work in particular contexts and thus, support implementation planning beyond the trial setting [12].

Aims

The aims of the process evaluation are to:

1. Assess implementation fidelity and understand how the intervention was implemented

2. Identify key contextual factors that impact intervention delivery and outcomes

3. Understand perceptions of different stakeholders about effectiveness, acceptability, and scalability of intervention components

4. Identify key facilitators and barriers in implementation of the intervention, and

5. Explain any adaptations to the intervention or intervention refinement during the study and their possible impact on the outcomes

METHODS

Conceptual Framework

The process evaluation will be guided by the MRC Framework for developing and evaluating complex interventions [13, 14]. The framework highlights the importance of examining the *implementation*, the *mechanism of impact* and their interaction with *contextual factors* to better understand how and why an intervention does or does not work. A recent update to the framework recognises that complex interventions have several phases including intervention development, feasibility assessment, implementation and evaluation, which may not always be sequential [15]. It recommends six core areas of inquiry at each phase before moving on to the next phase. They include (1) the intervention and its interaction with context; (2) the programme theory; (3) ways of engaging with diverse

For peer review only - http://bmjopen.bmj.com/site/about/guidelines.xhtml

stakeholders; (4) ways of intervention refinement; (5) identifying key uncertainties; and (6) economic considerations. The process evaluation will focus on the first five areas of inquiry, and the overall objectives have been framed to address these key areas.

Study Setting

The ARTEMIS cRCT will be implemented in 60 slum clusters across two cities New Delhi and Vijayawada in India. In each city 30 slum clusters will be included. New Delhi is a metropolis and one of the largest cities in India with a population of about 17 million and an estimated slum population of about 2 million [16,17]. Vijayawada is one of the largest cities in the state of Andhra Pradesh with an urban population of over 1.0 million[18,19] and an estimated slum population is estimated to be about 0.5 million[17]. The most widely spoken language in Delhi is Hindi, while in Vijayawada it is Telugu.

Patient and Public Involvement

Adolescent Expert Advisory Groups (AEAG) have been formed in each site. This group was involved throughout the intervention development phase in providing inputs and suggestions. Their contributions were critical for the co-creation of the anti-stigma content for adolescents. During the formative phase 34 meetings were held where the AEAG provided valuable feedback on ways to engage adolescents in the anti-stigma campaign.

Study Design

The study will use a mixed method, multiple case study design. Each slum cluster will constitute a 'case' for the study. A total of six cases or clusters will be purposively selected based taking a maximum variation approach. Slums will be purposively selected to represent different contexts, coverage and reach of intervention as well as the ease or difficulty of implementing the intervention.

Intervention Description

The ARTEMIS cRCT will test an intervention to address depression, increased risk of self-harm/suicide, or other significant emotional or medically unexplained complaints among adolescents living in urban slums in two cities in India. ARTEMIS has two components. The first is a campaign that aims to reduce stigma related to mental health and improve attitudes and behaviours towards adolescents with depression or at increased risk of self-harm/suicide. The second is a technology-enabled mHealth platform with an integrated electronic decision support system (EDSS), to help primary care doctors and NPHWs to diagnose and treat adolescents at high-risk of depression, self-harm, or suicide.

Before randomization and before the start of the intervention, a team of trained field investigators will screen adolescents at high-risk of depression or suicide using Patient Health Questionnaire- 9

For peer review only - http://bmjopen.bmj.com/site/about/guidelines.xhtml

(PHQ-9), which is a standardised psychometric tool for screening depression and suicide risk in the community [20, 21]. Adolescents who obtain a PHQ 9 score of ≥ 10 and/or a score of ≥2 to the suicide risk question on the PHQ-9 will be deemed as 'high-risk'. Due to the time delay between screening and randomisation and potential natural remission, a second screening will be carried out for adolescents identified at 'high-risk' before the baseline is administered. This process will help identify the final list of adolescents at 'high-risk' in all the clusters. The following information will be collected from the study cohort by the trained field investigators: socio-demographic characteristics, history of mental illness, treatment history, co-morbid conditions, stressful events experienced in the previous year, resilience, knowledge attitude and behaviours related to mental health and stigma associated with help seeking. A detailed protocol of the trial has been published [22].

A theory of change model for the intervention was developed with key stakeholders during the formative phase of the study in 2021. The logic model for intervention has been provided (Figure 1)

(Figure 1. Logic model about here)

Intervention Arm

In the intervention arm, the two components (i) the anti-stigma campaign and (ii) the technology-enabled mHealth based EDSS for screening, diagnosis and management of depression and self-harm will be delivered. (Figure 1).

Anti-stigma campaign

The anti-stigma campaign will include several Information Education and Communication (IEC) materials containing key messages around addressing mental health stigma, myths related to mental health, highlighting major stressors and their impact on mental health of adolescents and the importance of help seeking for mental health problems. The IEC materials will include posters, pamphlets, brochures, videos of persons with lived experience (PWLE), animation videos, audio dramas, games, magic shows, rallies, and street plays. The materials will be co-created with adolescents from the study sites. Adolescent Expert Advisory Groups (AEAGs) will be created in both locations consisting of adolescents from the community, with the purpose of involving them on working with the implementation team to co-create the anti-stigma campaign. Details of the process of forming AEAGs and their engagement in shaping the anti-stigma campaign has been published elsewhere [23].

Peer-educators

For peer review only - http://bmjopen.bmj.com/site/about/guidelines.xhtml

Based on findings from the formative phase of the study, peer groups were established comprising of adolescents from the community in the age group 13-19 years, some of whom were from the study cohort. Their role will be to provide support to adolescents who approached them to discuss problems and to promote mental health awareness in the community. All the members of the peer groups will be provided basic training by the research team on mental health, safe practices while using social media, addiction and substance use and promoting mental wellbeing through engaging in pleasurable activities such a listening to music, sharing problems with trusted individuals, having friends, exercising, and eating healthily.

Technology-enabled EDSS

The EDSS platform has been developed to assist primary care doctors and NPHWs to screen, diagnose and manage depression, other significant emotional or medically unexplained complaints and suicide risk among adolescents. High-risk adolescents will be referred to the doctor electronically via the mHealth platform by the NPHWs. In each cluster, one doctor and several NPHWs will be trained. The NPHWs will be trained to use the EDSS to provide basic supportive advice to adolescents at high-risk of depression or self-harm, refer them to the primary care doctors, and track treatment advise given by the doctors and will follow-up for treatment adherence. The NPHWs will make regular home visits to encourage adolescents at high risk and their families to seek help from the doctor. The NPHWs will also use the EDSS to clinically monitor the severity of high-risk individuals at four months and at ten months after the start of the intervention. High-risk individuals will be provided referral cards to take to the primary care doctors. The primary care doctors will be trained to use an EDSS to clinically diagnose and manage adolescents with depression, other significant emotional or medically unexplained complaints and self-harm/suicide ideation based on WHO's mhGAP-Intervention Guide algorithm[24]. Adolescents needing specialist care will be referred to a psychiatrist in a government facility. The EDSS will also capture and store (on a cloud server) information regarding adolescents' visits and type of care provided by the primary care doctor-counselling/pharmacotherapy/combination of both/referral/follow-up. All data will be encrypted and protected using user-defined passwords. The information will be visible to the NPHWs, the project supervisors and research team only. A colour-coded traffic light system integrated in the application will assist the NPHWs to identify high-risk adolescents who need to be prioritised for followed up. The EDSS will also provide them with simple questions tailored to the priority level as indicated by the traffic-light coding system, that they can ask the adolescents during follow-up to ensure appropriate treatment adherence or follow-up with doctors or mental health specialists.

Control Arm

For peer review only - http://bmjopen.bmj.com/site/about/guidelines.xhtml

The control arm will receive enhanced usual care that will include general psychoeducation through pamphlets to raise awareness on common mental disorder in adolescents amongst the wider community. Parents/guardians of adolescents identified at high-risk will be advised to consult a primary care doctor or mental health professional. Those with high scores on PHQ-9 (a score of >=15 on PHQ9) and/or at high risk of suicide (suicide score >=2), indicating severe depression and/or high risk of suicide will be asked to seek care immediately and will be given a list of health facilities (with contact details) where such treatment is available. All NPHWs will be asked to follow up with the adolescents and their families more intensively and will recommend that they visit a doctor/psychiatrist immediately.

Data Collection

Quantitative data for the process evaluation will draw on metrics collected sourced from the open-source medical record system (OpenMRS; (OpenMRS.org). The OpenMRS, is a standardized community-driven open-source software for storing and processing medical record information Quantitative data will be collected throughout the intervention via the mHealth platform. This will include data on screening outcome, treatment seeking, follow ups, and treatment provided for the high-risk cohort. Data will also be used to understand usage patterns of the EDSS by primary care doctors and NPHWs. The mHealth platform will capture this data in tablets used by NPHWs and primary care doctors for screening, treatment, and follow-up of the high-risk cohort. This data will be utilised to assess reach, effectiveness, and service utilisation. Data on competency and fidelity measures will also be captured throughout the intervention.

Qualitative data collection will include key informant interviews (KII) and focus group discussions (FGDs) with various stakeholders including high-risk and non-high-risk cohort in both arms, parents, primary care doctors, NPHWs, members of the AEAG and peer leaders. A total of 33 FGDs and 28 interviews are planned across the six purposively selected slum clusters equally divided between both sites. This will include four intervention clusters (two each in Delhi and Vijayawada) and 2 control clusters (one each in Delhi Vijayawada) (Table 1). The KIIs and FGDs with stakeholders will help capture information related to fidelity of the intervention and identify any gaps that could be addressed. Written informed consent will be sought from all participants selected for KIIs and FGDs.

(Table 1 about here)

Data Analysis

The conceptual framework for the study and how it integrates with the key parameters from the MRC frameworks is provided in Table 2.

For peer review only - http://bmjopen.bmj.com/site/about/guidelines.xhtml

(Table 2 about here)

The case study methodology will also be used as it is recognised to be suitable to study complex interventions in different contexts and helps to capture ‘the complexity of the case, the relationship between the intervention and the context and how the intervention worked (or did not)’ ([25]. Following Pfadenhauer et al (2016) we define context as ‘a set of characteristics and circumstances that consist of active and unique factors that surround the implementation. As such it is not a backdrop for implementation but interacts, influences, modifies and facilitates or constrains the intervention and its implementation’ [26]. We will be selecting both intervention and control sites as cases to avoid the Hawthorne effect. Two slum cluster in the control arm will also be selected for the case study to enable comparison, to understand contextual factors and any changes to usual care in the absence of an intervention. The importance of studying changes in control arms has been recognised as being useful in understanding intervention impact [27]. . A multiple case study design will be use where each case will be analysed at the case level, but we will also pull together all the cases from the intervention and control arms for a more in-depth analysis and comparison. Basic descriptive analysis will be conducted with the quantitative data. Qualitative data will be transcribed by a professional vendor and the transcripts will be read by at least two members of the research team and coded. Coding will be carried out using a priori codes based on the conceptual framework (Table 3) with the help of N-Vivo 12 software.

(Table 3 about here)

Any additional codes emerging during the analysis will be added to the coding framework. Members of the research team will read transcripts separately and come up with codes. The research team will then compare and combine their codes to evaluate their fit and usefulness and will examine the differences in code to see if any new insights can be generated. The code list will be finalised after a discussion between the researchers to establish agreement among coders. Qualitative and quantitative data will be triangulated to arrive at a comprehensive understanding of the intervention. Triangulation helps in confirming the findings from quantitative and qualitative data thereby increasing validity as well as leading to a better understanding of phenomena being studied [28]

Assessment of Implementation outcomes

Implementation fidelity refers to the extent to which an intervention was implemented as intended. The RE-AIM [29] (Reach, Efficacy, Adoption, Implementation and Maintenance) framework will be applied to understand implementation outcomes. Additionally, the APEASE (Affordability, Practicability, Effectiveness and cost-effectiveness, Acceptability, Safety/Side Effects and , Equity)

For peer review only - http://bmjopen.bmj.com/site/about/guidelines.xhtml

criteria for evaluation of behaviour change interventions [30] will be utilised [31,32]. The framework has some similarities with RE-AIM, but includes two additional parameters, i.e., practicability and safety. An intervention is practicable if 'it can be delivered as designed through the means intended to the target population'[30].

We will assess fidelity in 3 components (a) delivery of anti-stigma campaign, (b) implementation of the mHealth component, including trainings. Indicators have been developed for all these components to measure frequency of exposure and coverage. The quality of training provided to primary doctors on the mhGAP, NPHWs on the priority listing app and peer educators on promoting mental wellbeing and competency will be assessed through various tools including a post training satisfaction survey, pre-post-test self-assessment checklists as well as though rating by trainers and staff on competency checklists.

Discussion

This paper describes the design of a mixed method process evaluation for the ARTEMIS cRCT, which involves an anti-stigma campaign and a mobile device-based decision support system for primary care doctors and non-physician health workers, to improve treatment of adolescents at high-risk of depression, other significant emotional or medically unexplained complaints and suicide risk.

The process evaluation will help us understand and explain key causal mechanisms that led to change. and will therefore strengthen the understanding of the implementation process by highlighting various barriers and facilitators. Furthermore, it will provide an understanding of how local context played a role in the way the intervention was implemented and help identify the need and impact of any adaptations made to the intervention. The process evaluation will provide stakeholder perspectives on aspects of the intervention that worked and those that need further adaptation.

Strengths and Limitations

The protocol was developed using existing implementation science theories and frameworks and combines qualitative and quantitative measures to understand key aspects of how the intervention was implemented and which aspects worked or need further improvement.

Ethics and Dissemination

The study has received formal ethics approval from the Ethics Committee of the George Institute for Global Health India on 04 September 2020 (Project number 17/2020). The study also received formal ethics approval from the Research Governance and Integrity Team, Imperial College, London on 8th June 2022 (ICREC Reference number: 22IC7718). The Health Ministry's Screening Committee (HMSC),

For peer review only

For peer review only - http://bmjopen.bmj.com/site/about/guidelines.xhtml

Indian Council for Medical Research (ICMR) has also provided approval to the project (ID 2020-9770). Findings will be disseminated to study participants and other stakeholders at a policy symposium. All identifiable personal data will be stored in password protected secured servers located at The George Institute for Global, India office in Hyderabad. Only deidentified data will be disseminated. Data will be available with the PI on an accessible data repository, which can be accessed by other researchers, subject to a formal request to the PI to access the data for research purposes.

Trial Status

At the time of writing the paper, the intervention had started in both sites. Randomization was executed in Vijayawada and in Delhi on 12th December 2022. Intervention components were implemented in both the sites.

References

1. UNICEF: On My Mind: The State of the World's Children 2021. New York: United Nations Children's Fund; 2021. Available from: https://www.unicef.org/media/114636/file/SOWC-2021-full-report-English.pdf
2. Bor W, Dean AJ, Najman J, Hayatbakhsh R: Are child and adolescent mental health problems increasing in the 21st century? A systematic review. The Australian and New Zealand journal of psychiatry. 2014.48(7):606–16. Available from: https://pubmed.ncbi.nlm.nih.gov/24829198/
3. Gururaj G, Varghese M, Benegal V, Rao GN, Pathak K, Singh LK, Mehta RY, Ram D, Shibukumar TM, Kokane A, Lenin Singh RK, Chavan BS, Sharma P, Ramasubramanian C, Dalal PK, Saha PK , Deuri SP, Giri AK, Kavishvar AB, Sinha VK, Thavody J, Chatterji R, Akoijam MR: National Health Survey of India 2015-16: Prevalence, patterns and outcomes. Bangalore; 2016.
4. Joshi R, Alim M, Maulik PK, Norton R: A contemporary picture of the burden of death and disability in Indian adolescents: data from the Global Burden of Disease Study. International Journal of Epidemiology. 2017.46(6):2036. Available from: /pmc/articles/PMC5837726/
5. Patton GC, Sawyer SM, Santelli JS, Ross DA, Afifi R, Allen NB, Arora M, Azzopardi P, Baldwin W, Bonell C, Kakuma R, Kennedy E, Mahon J, McGovern T, Mokdad AH, Patel V, Petroni S, Reavley N, Taiwo K, Waldfogel J, Wickremarathne D, Barroso C, Bhutta Z, Fatusi AO, Mattoo A, Diers J, Fang J, Ferguson J, Ssewamala F, Viner RM: Our future: a Lancet commission on

For peer review only

adolescent health and wellbeing. Lancet (London, England). 2016.387(10036):2423. Available from: /pmc/articles/PMC5832967/

6. Culbreth R, Swahn MH, Ndetei D, Ametewee L, Kasirye R: Suicidal Ideation among Youth Living in the Slums of Kampala, Uganda. International Journal of Environmental Research and Public Health 2018, Vol. 15, Page 298. 2018.15(2):298. Available from: https://www.mdpi.com/1660-4601/15/2/298/htm

7. Subbaraman R, Nolan L, Shitole T, Sawant K, Shitole S, Sood K, Nanarkar M, Ghannam J, Betancourt TS, Bloom DE, Patil-Deshmukh A: The psychological toll of slum living in Mumbai, India: a mixedmethods study. Social science & medicine (1982). 2014.119:155. Available from: /pmc/articles/PMC4252879/

8. Mehra, D., Lakiang, T., Kathuria, N., Kumar, M., Mehra, S., & Sharma, S. (2022). Mental Health Interventions among Adolescents in India: A Scoping Review. *Healthcare (Basel, Switzerland)*, *10*(2), 337. https://doi.org/10.3390/healthcare10020337

9. Mathias, K., Pandey, A., Armstrong, G. *et al.* Outcomes of a brief mental health and resilience pilot intervention for young women in an urban slum in Dehradun, North India: a quasi-experimental study. *Int J Ment Health Syst* **12**, 47 (2018). https://doi.org/10.1186/s13033-018-0226-y

10. Leventhal, Katherine Sachs et al. "Building psychosocial assets and wellbeing among adolescent girls: A randomized controlled trial." *Journal of adolescence* vol. 45 (2015): 284-95. doi:10.1016/j.adolescence.2015.09.011

11. Gonsalves, Pattie P et al. "App-based guided problem-solving intervention for adolescent mental health: a pilot cohort study in Indian schools." *Evidence-based mental health* vol. 24,1 (2021): 11-18. doi:10.1136/ebmental-2020-300194

12. Lockwood I., Walker RM, Latimer S., Chaboyer W., Cooke M., Gillespie BM. Process evaluations undertaken alongside randomised controlled trials in the hospital setting: A scoping review. Contemporary Clinical Trials Communications, Volume 26, 2022, 100894,ISSN 2451-8654, https://doi.org/10.1016/j.conctc.2022.100894

13. Moore GF, Audrey S, Barker M, Bond L, Bonell C, Hardeman W, Moore L, O'Cathain A, Tinati T, Wight D, Baird J: Process evaluation of complex interventions: Medical Research Council guidance. BMJ (Online). 2015.350.

For peer review only

14. Craig P, Dieppe P, Macintyre S, Mitchie S, Nazareth I, Petticrew M: Developing and evaluating complex interventions: The new Medical Research Council guidance. BMJ. British Medical Journal Publishing Group; 2008. p. 979–83. Available from: www.mrc.ac.uk/

15. Skivington K, Matthews L, Simpson SA, Craig P, Baird J, Blazeby JM, Boyd KA, Craig N, French DP, McIntosh E, Petticrew M, Rycroft-Malone J, White M, Moore L: A new framework for developing and evaluating complex interventions: update of Medical Research Council guidance. BMJ. 2021.374. Available from: https://www.bmj.com/content/374/bmj.n2061

16. Office of the Registrar General & Census Commissioner India: Primary Census Abstract Data Tables. [accessed 2022 Jun 29]. Available from: https://web.archive.org/web/20190714165104/http://censusindia.gov.in/pca/pcadata/pca.html

17. Ministry of Housing and Urban Poverty Alleviation Government of India: Slums in India : A Statistical Compendium 2015. 2015 [accessed 2022 Jul 19]. Available from: http://nbo.nic.in/pdf/SLUMS_IN_INDIA_Slum_Compendium_2015_English.pdf

18. Government of Andhra Pradesh: Handbook of Statistics 2019: Krishna District. 2019.

19. Office of the Registrar General & Census Commissioner: Population Finder 2011. Ministry of Home Affairs,Government of India [Internet]. Available from: https://censusindia.gov.in/census.website/data/population-finder

20. Pfizer Inc.: Patient Health Questionnaire- 9 (PHQ-9). 2010 [accessed 2020 Jul 20]. Available from: https://www.phqscreeners.com/images/sites/g/files/g10060481/f/201412/PHQ9_English for India.pdf

21. Kroenke K, Spitzer RL, Williams JBW: The PHQ-9: Validity of a brief depression severity measure. Journal of General Internal Medicine. 2001.16(9):606–13. Available from: /pmc/articles/PMC1495268/?report=abstract

22. Yatirajula SK, Kallakuri S, Paslawar S, Mukherjee A, Bhattacharya A, Chatterjee S, Sagar R, Kumar A, Lempp H, Raman U, Singh R, Essue B, Billot L, Peiris D, Norton R, Thornicroft G, Maulik PK: An intervention to reduce stigma and improve management of depression, risk of suicide/self-harm and other significant emotional or medically unexplained complaints among adolescents living in urban slums: protocol for the ARTEMIS project. Trials 2022 23:1.

For peer review only

For peer review only - http://bmjopen.bmj.com/site/about/guidelines.xhtml

2022.23(1):1–15. Available from: https://trialsjournal.biomedcentral.com/articles/10.1186/s13063-022-06539-8

23. Yatirajula SK, Kallakuri S, Paslawar S, Mukherjee A, Khan NA, Kumari K, Sagar R, Thornicroft G, Maulik PK: Engaging with adolescents to navigate the Adolescents' Resilience and Treatment nEeds for Mental health in Indian Slums (ARTEMIS) trial. Journal of Global Health.

24. WHO: mhGAP Intervention Guide for mental, neurological and substance use disorders in non- specialized health settings (Version 1.0). Geneva; 2010 [accessed 2020 Jun 11]. Available from: www.who.int/mental_health/mhgap

25. Yin R. Case study research and applications: design and methods. Los Angeles: Sage Publications Inc; 2018

26. Pfadenhauer L, Rohwer A, Burns J, Booth A, Lysdahl KB, Hofmann B, Gerhardus A, Mozygemba K, Tummers M, Wahlster P, et al. Guidance for the assessment of context and implementation in health technology assessments (HTA) and systematic reviews of complex interventions: the Context and Implementation of Complex Interventions (CICI) framework: Integrate-HTA; 2016

27. Rachel Jewkes, Andrew Gibbs, Esnat Chirwa & Kristin Dunkle (2020) What can we learn from studying control arms of randomised VAW prevention intervention evaluations: reflections on expected measurement error, meaningful change and the utility of RCTs, Global Health Action, 13:1, DOI: 10.1080/16549716.2020.1748401

28. Bekhet Ak., Zauszniewski JA. Methodological triangulation: an approach to understanding data. Nurse Researcher. 20, 2, 40-43. doi: 10.7748/nr2012.11.20.2.40.c9442

29. Waitzkin H: Different Views A Marxian Interpretation of the Growth And Development of Coronary Care Technology. American Journal of Public Health. 1979.69(12):1260–8. Available from: http://ajph.aphapublications.org/doi/pdf/10.2105/AJPH.69.12.1260

30. Michie S, Atkins L, Gainforth HL: Changing Behaviour to Improve Clinical Practice and Policy. In: Gonçalves A, Azevedo A, Lobo F, editors. Novos Desafios, Novas Competências: Contributos Atuais da Psicologia. Braga,Portugal: Axioma - Publicações da Faculdade de Filosofia; 2016. p. 41–60.

31. Michie S, West R, Rogers MB, Bonell C, Rubin GJ, Amlôt R: Reducing SARS-CoV-2 transmission in the UK: A behavioural science approach to identifying options for increasing

For peer review only

For peer review only - http://bmjopen.bmj.com/site/about/guidelines.xhtml

adherence to social distancing and shielding vulnerable people. British Journal of Health Psychology. 2020.25(4):945–56.

32. Brierley ML, Smith LR, Bailey DP, Ojo SO, Hewson DJ, Every SA, Staines TA, Chater AM: Evaluating a multi-component intervention to reduce and break up office workers' sitting with sit-stand desks using the APEASE criteria. BMC Public Health. 2022.22(1):1–15. Available from: https://bmcpublichealth.biomedcentral.com/articles/10.1186/s12889-022-12794-w

Author statement

The paper was conceptualised by PKM. AM wrote the original draft with contributions from SKY. PKM commented on multiple drafts before sending a prefinal version to everyone listed as authors. SK, SP, HL, UR, BME, RS, ReS, DP, RN, GT reviewed the draft and provided critical intellectual inputs and comments to the draft. PKM leads the implementation of the trial in India along with SKY, SK, SP and AM. SKY, SK and SP played a key role in implementing research activities in the sites. All authors read and approved the final manuscript.

Funding

Adolescents' Resilience and Treatment nEeds for Mental health in Indian Slums (ARTEMIS) is funded by UK Research and Innovation/Medical Research Council (UKRI/MRC), Grant no: MR/S023224/1. SKY, SK, and SP are all partially or fully supported by ARTEMIS. PKM and AM are partially supported through NHMRC/GACD grant (SMART MentalHealth-APP1143911) and ARTEMIS. PKM is the PI for ARTEMIS and Co-PI for SMART Mental Health. HL and GT are partly support by ARTEMIS. GT is also supported by the National Institute for Health Research (NIHR) Applied Research Collaboration South London at King's College London. The views expressed are those of the author(s) and not necessarily those of the NHS, the NIHR or the Department of Health and Social Care. GT is also supported by the MRC-UKRI in relation to the Indigo Partnership (MR/R023697/1) awards. The funding agency had no role in the writing of the manuscript or the decision to submit it for publication.

Competing interests statement

We declare that the George Institute for Global Health has a part-owned social enterprise, George Health Enterprises, which has commercial relationships involving digital health innovations.

Acknowledgements

We acknowledge the contribution of all members of the Adolescent Expert Advisory Group in Delhi and Vijayawada who has generously contributed their time and expertise to the development of ARTEMIS. GT

For peer review only

is supported by the National Institute for Health and Care Research (NIHR) Applied Research Collaboration South London (NIHR ARC South London) at King's College Hospital NHS Foundation Trust. The views expressed are those of the author(s) and not necessarily those of the NIHR or the Department of Health and Social Care.

Data Statement Section

This is a process evaluation protocol paper; no data has been collected as yet and therefore there is no data that can be shared. However once the process evaluation is completed, the data will be made available on a data repository.

For peer review only

Table 1: Qualitative Data Collection Plan

**Intervention Arm**		
Data Sources	**FGDs Planned**	**Interviews planned**
Non-physician Health Workers (NPHWs)	4	-
Adolescents from high-risk cohort	8	-
Adolescents from nonhigh-risk cohort	4	
Parents of high-risk adolescents in the study cohort		24
Members of Adolescent Expert Advisory Groups (AEAG)	5	-
Peer Leaders	4	-
Field Staff	2	-
Key implementation team members	1	
Primary Care Doctors	-	4
**Control Arm**		
Non Physician Health Workers (NPHWs)	2	-
Adolescents from study cohort	4	-
TOTAL	34	28

For peer review only - http://bmjopen.bmj.com/site/about/guidelines.xhtml

Table 2. Conceptual Framework for Process Evaluation

Broad Area of Enquiry	Domains of Inquiry	Key Questions/Process Measures	Data Source
**CONTEXT**	Differences in context	• What are the differences in terms of geography, slum conditions, socio-cultural norms, economic between the sites and among the clusters?<br>• What are the contextual factors which affect programme delivery, stakeholder response and intervention uptake in different settings?<br>• Who were the key stakeholders who could play a role in improving intervention outcomes?	Secondary data;<br>Formative research data<br>FGDs with project staff<br>FGDs with key members of the implementation team
	Intervention adaptions based on context	• What are some of the key contextual factors which influenced the overall implementation of the intervention?<br>• What were some of the context specific adaptations that were made to address emerging challenges?	FGDs with project staff<br>FGDs with key members of the implementation team<br>Project documentation completed during the project
	Contextual Barriers and Facilitators	• What are key facilitators in different contexts that helped in intervention delivery and uptake?	FGDs with project staff

For peer review only - http://bmjopen.bmj.com/site/about/guidelines.xhtml

		• What are the barriers in various contexts for implementing the intervention components?	FGDs with key members of the implementation team

**IMPLEMENTATION**	Implementation fidelity	Was the intervention delivered as it was planned?	Program records and documents; Observation and rating
	Intervention Reach	• What was the coverage of the different *anti-stigma campaign* methods, in terms of:<br>- Total persons reached (including age and gender-wise break-up)<br>- Slums and clusters covered.<br>- Number and proportion of high-risk cohort reached.<br>- Number and proportion of non-high-risk cohort reached.<br>- Key stakeholders reached.<br>• What was the reach of the mHealth services:	Project records and documents<br><br>Backend data<br><br>FGDs with project staff<br><br>FGDs with key members of the implementation team<br><br>Interviews with NPHWs

For peer review only - http://bmjopen.bmj.com/site/about/guidelines.xhtml

		- Number and proportion of high-risk cohort in the intervention arm provided counselling or follow-up services by NPHWs.<br>- Number and proportion of high-risk cohort from the intervention arm who sought care with the primary care doctor.<br>- Number and proportion high risk-cohort from the control arm who sought care.<br><br>• Did the NPHWs face any challenge in reaching out adolescents and their families during the intervention?	
	Intervention effectiveness	• What was the perception of adolescents and key stakeholders about the utility effectiveness of the anti-stigma campaign?<br>• What are some of the key take home messages from anti stigma campaign for adolescents and community members?<br>• What was the perception of NPHWs about impact of anti-stigma campaign in their community?	Community satisfaction survey done at the end of street play performance.<br><br>Outcome survey data;<br>Backend data<br><br>FGDs with adolescents<br><br>FGDs with AEAG and peer leaders

For peer review only - http://bmjopen.bmj.com/site/about/guidelines.xhtml

		• What is the association between exposure to anti stigma content with changes in KAB scores and care seeking?<br>• What is the perception of NPHWs about effectiveness of mHealth based EDSS for management of depression and self-harm in adolescents?<br>• What is the perception of doctors about effectiveness of mHealth based EDSS for community-based management of depression and self-harm in adolescents?	Interviews with parents
	Intervention acceptability and adoption	• What was the experiences of NPHWs in using EDSS for providing care (challenges, perceived benefits, potential for routine use of mHealth)?<br>• What was the experiences of doctors about using EDSS for providing care (challenges, perceived benefits, potential for routine use of mHealth)?<br>• What were some key features of use of EDSS by doctors:	Back-end data<br>FGDs with NPHWs<br>Interviews with doctors<br>Interview with high-risk adolescents and their parents

For peer review only

For peer review only - http://bmjopen.bmj.com/site/about/guidelines.xhtml

		- Average time taken for diagnosis and identification of treatment plan using mhGAP over time.<br>- Association between type of CMD and time taken for diagnosis and identification of treatment plan using mhGAP.<br><br>• What were the perceptions of high-risk adolescents and their parents about ease of getting treatment through mHealth?	
	Practicability	• What were the challenges/barriers in delivering of the different strategies of anti-stigma campaign to adolescents?<br>• What were the challenges of the electronic decision support systems by NPHWs and doctors for diagnosis and management of depression and self-harm among adolescents?<br>• Doctor's experiences of interactions with adolescents<br>• Experiences of NPHWs with adolescents during follow up and motivating them and their families to visit the doctor initially and for follow up	FGDs with NPHWs<br><br>Interviews with doctors<br><br>FGDs with project staff

For peer review only - http://bmjopen.bmj.com/site/about/guidelines.xhtml

	Safety and Side Effects	• Were there any unanticipated negative consequences because of the anti-stigma campaign?<br>• Were there any safety concerns related to using mHealth based EDSS platform for management of depression and self-harm among adolescents?	FGDs with adolescents<br>FGDs with NPHWs<br>Interviews with doctors<br>FGDs with project staff

**MECHANISM OF IMPACT**	Variation in outcomes	What kind of cluster level variation is observed in in the outcomes? What works, for whom and in what context?	Outcome data<br>Backend data;<br>FGDs with NPHWs<br>Interviews with doctors<br>FGDs with project staff
	Unexpected outcomes	What are some unexpected outcomes and what factors can be attributed to them?	Outcome data<br>Back-end data;<br>FGDs with NPHWs<br>Interviews with doctors<br>FGDs with project staff

For peer review only

For peer review only - http://bmjopen.bmj.com/site/about/guidelines.xhtml

	Theory of Change (ToC)/ Pathways to achieving outcomes	What were some of the key pathways to improved outcomes for high-risk adolescents?<br>What changes and adaptations were made to the ToC during the intervention?<br>What were some of the key assumptions regarding causal mechanisms that need to be modified?	Outcome data<br>Back-end data<br>Project documents<br>FGDs with project staff

For peer review only

Table 3: Qualitative Data Collection Areas of Inquiry

Type of Group/Individual	Some areas of inquiry
Non-Physician Health Workers	• Perceptions on effectiveness and appropriateness of training received.<br>• Experience of using mHealth platform for delivering the intervention including challenges or facilitators<br>• Perception about effectiveness anti-stigma campaign in improving help seeking.<br>• Facilitators and barriers in treatment seeking by high-risk cohort.<br>• Overall experience of participating in the trial.<br>• Perception about any personal benefits/harms due to their association with the project.<br>• Perception about what role they see for themselves in the community once the project comes to an end.<br>• Any spillover effects-experiences of helping or sharing information with other adolescents not in the study cohort.
Project Field staff in each site	• Barriers or facilitators in delivering anti-stigma campaign in the community.<br>• Barriers and facilitators in delivering mHealth component.<br>• Perceived factors which explain high/low treatment seeking in different clusters.<br>• Key lessons learnt and suggestions for implementation of intervention components.<br>• Perceptions on training appropriateness, effectiveness and methods.

For peer review only - http://bmjopen.bmj.com/site/about/guidelines.xhtml

Study participants from high-risk cohort in intervention arm	• Perception appropriateness of anti-stigma content and methods for adolescents.<br>• Perceptions about impact and effectiveness of anti-stigma campaign in community.<br>• Any reported change in perception about mental health related stigma and help seeking in self or family.<br>• Perception about case detection, treatment and follow-up through EDSS.<br>• Facilitators and barriers in help-seeking by adolescents<br>• Experience of care and perception about quality of care, provided by doctors.<br>• Positive/negative experiences as a study participant.<br>• Perception about benefits/effectiveness of the intervention.<br>• Any spillover effects-experience of helping or sharing information with other adolescents not in the study cohort.
Study participants from non-high-risk cohort in intervention arm	• Perceptions about impact and effectiveness of anti-stigma campaign in community.<br>• Any reported changes in perception about mental health related stigma and help seeking in self or family.<br>• Facilitators and barriers in treatment seeking.<br>• Positive/negative experiences as a study participant.<br>• Perception about benefits/effectiveness of the intervention.<br>• Any spillover effects-experience of helping or sharing information with other adolescents not in the study cohort.
Study participants from (high-risk and non-high-risk	• Perception about impact and effectiveness of anti-stigma content.<br>• Any reported change in perception about mental health related stigma and help seeking in self or family.<br>• Facilitators and barriers in help seeking.

For peer review only

cohort) in the control arm	• Experience as a study participant.
Parents of adolescents in the study cohort	• Perception about impact and effectiveness of anti-stigma content.<br>• Any reported change in perception about mental health related stigma and help seeking in self or family.<br>• Knowledge about key stressors and symptoms of mental health problems in adolescents.<br>• Facilitators and barriers in help-seeking by adolescents.<br>• Experience of help seeking.<br>• Perception about benefits/effectiveness of the intervention.
Adolescent expert Advisory Groups in each site	• Perception about their role on co-creation of anti-stigma material and feedback on the whether the anti-stigma material was able reflect their suggestions.<br>• Perception about impact and effectiveness of anti-stigma content among adolescents and community members.<br>• Any reported change in perception about mental health related stigma and help seeking in self or family.<br>• Perceived personal benefit/challenges in being an AEAG member.<br>• Perception about benefit and effectiveness of mHealth component.<br>• Facilitators and barriers in help seeking by high-risk adolescents.<br>• Suggestions on improving engagement/involvement of younger adolescents in AEAGs and the co-creation process.

For peer review only

Peer Group - Intervention arm	• Perceptions on effectiveness and appropriateness of training received.<br>• Perceptions about impact and effectiveness of anti-stigma campaign in community.<br>• Perception about effectiveness anti-stigma campaign in improving help seeking by adolescents.<br>• Facilitators and barriers in treatment seeking by high-risk cohort.<br>• Experience if any of providing help to adolescents in the community who reached out to the peer group.<br>• Overall experiences of participating in the trial.<br>• How does the learnings from the project/trainings will be useful beyond the project period.
Doctors from intervention arm	• Perceptions on effectiveness and appropriateness of training received.<br>• Experience of using technology-based decision support system for diagnosis and management of depression and self-harm among adolescents.<br>• Perceived effectiveness anti-stigma campaign.<br>• Possible facilitators and barriers to scaling up mHealth component.<br>• Overall experience of participating in the trial including any challenges.<br>• Facilitators and barriers in help seeking and treatment adherence among adolescents.<br>• Whether learnings from the project/trainings will be useful beyond the project period.
Study participants from high-risk cohort in intervention arm who visited the	• Experience of seeking care from primary care doctor and perception about quality of care.<br>• Level of comfort/discomfort in sharing their mental distress experiences with doctors.<br>• Perceived benefit, if any, as a result of treatment.<br>• Barriers and facilitators for seeking treatment from a specialist (in case of referral).<br>• Views about help-seeking in future or continuing care if advised by the doctor.

For peer review only - http://bmjopen.bmj.com/site/about/guidelines.xhtml

doctor and were given medical and/or psychological treatment.	• Positive/negative experiences as a study participant.<br>• Suggestions for the programme in the future.
Parents/guardians of study participants from high-risk cohort in intervention arm who visited the doctor	• Perception about impact and effectiveness of anti-stigma content.<br>• Any reported change in perception about mental health related stigma and help seeking in self or family.<br>• Knowledge about key stressors and symptoms of mental health problems in adolescents.<br>• Knowledge perception about mental health problems of their ward.<br>• Barriers and facilitators for seeking mental healthcare.<br>• Experience of seeking mental health care for their wards.<br>• Perceived benefit, if any, as a result of treatment.<br>• Views about help-seeking in future or continuing mental health care if advised by the doctor.<br>• Suggestions for the programme in the future.

For peer review only

For peer review only - http://bmjopen.bmj.com/site/about/guidelines.xhtml

Input

- Funding
- Research and project staff
- Trainings
- Tablets and EDSS software
- IEC Material
- IT support
- Meetings with Adolescent Expert Advisory Group for co-creation of anti-stigma IEC

ASSUMPTIONS

- Mental health related help seeking among adolescents is low due to stigma and poor knowledge.
- Non physician health workers and doctors have limited knowledge about identification and management of depression and suicidal ideation/self-harm among adolescents .
- In resource poor settings like slums, availability of mental health services can improve access to care
- Trained primary level health workers can help in early identification, management and referral of adolescents needing mental health care.
- Task sharing through electronic decision support system (EDSS) is more efficient because it helps to cover the difficult to reach population and where trained mental health professionals are unavailable; helps real time tracking (accountability); helps linking different levels of health providers easily.

Activities

1. ***Anti Stigma Campaign using the following***
   a) Print material (brochures, pamphlets, posters)
   b) Videos with awareness message (video of person with lived experience (PWLE), animation video)
   c) Audio drama
   d) Interactive games with mental health messages (snakes and ladders, hopscotch, board games)
   e) Street Play (live performance and screening of recorded play)
   f) Magic show with mental health messages (live performance and screening of recorded show)
   g) Rallies
2. ***Training and capacity building of***
   a) Non Physician Health Workers (NPHWs)
   b) Primary care doctors
   c) Field investigators
   d) Peer Educators
3. **Technology enabled mental health service delivery**
   a) Community level screening for adolescents at high risk of depression and self-harm
   b) Treatment by primary level doctors using EDSS
   c) Follow-up of 'high risk' adolescents by NPHWs

Outputs

1. ***In the intervention slum clusters***
   a) All available adolescents in the intervention arm of the study cohort will be exposed to all forms of anti-stigma campaign material at least once during the intervention
   b) Peer leaders (young people) from the community will be identified who will assist in implementing the anti-stigma campaign.
2. ***Trained field staff/ and peer educators will***
   a) Trained field staff conduct community screen and baseline
   b) Trained field staff run anti-stigma campaign in the community
   c) Peer educators encourage adolescents to participate in anti-stigma activities
3. ***Technology enabled mental health delivery model will be used for service delivery in the following ways***
   a) Primary care doctors provide evidence-based care to high-risk adolescents seeking care using the EDSS
   b) NPHWs follow-up ≥ 80% high-risk adolescents in the in one year using 'priority- list' generated by M-Health platform to
   c) Symptom profile of all high-risk individuals in the intervention arm will be monitored using clinical data uploaded on the server

Outcomes

SHORT TERM

1. Improved knowledge, attitude and behaviour (KAB) related to mental health among adolescents.
2. Positive change stigma perception about help seeking among adolescents.
3. Increase in proportion of screen positive adolescents seeking care
4. NPHWS are able to follow up and refer cases of high-risk adolescents to the primary care doctor.
5. Primary care doctors have enhanced capacity to provide evidence-based care to adolescents with common mental disorders.

END OF INTERVENTION OUTCOMES

1. Improved task sharing and better linkage between ASHAs, doctors and specialists.
2. Larger proportion of people have improved symptom profile
3. Improved remission of high risk individuals.

Impact

- Reduction in stigma associated with depression and self-harm among adolescents, caregivers and communities .
- Programme components are incorporated in mental health programmes and policy nationally.
- Programme components are adapted/adopted in low resource settings

MECHANISM OF IMPACT

- EDSS will provide NPHWs and primary care doctors with easy tools for diagnosis and evidence-based care for adolescents at high risk of depression and self-harm
- Reduced stigma related to mental health will lead to improved perceptions about help seeking.
- Training would sensitise and build capacities of NPHWs and primary level doctors to provide care to adolescents who seek help for mental health.

CONTEXT *[Contextual factors which may affect the intervention are based on findings of the formative study, and field observations]*

- Adolescents living in slum communities are particularly vulnerable to mental health related problems due to living conditions and resource constraints. Violence, easy access to addictive substances, poor sanitation and waterlogging in the area add to the vulnerability.
- There are several demand side barriers for adolescents seeking help for mental health problems including limited knowledge and stigma.
- Several supply side barriers exist in accessing care at the primary care level such as lack of expertise to identify and manage adolescents with symptoms of common mental disorders.

EXTERNAL FACTORS

- Community unrest/riots
- Flooding
- Government approvals

SPIRIT

STANDARD PROTOCOL ITEMS: RECOMMENDATIONS FOR INTERVENTIONAL TRIALS

SPIRIT 2013 Checklist: Recommended items to address in a clinical trial protocol and related documents*

Section/item	Item No	Description
**Administrative information**		
Title	1	(Page 1)
Trial registration	2a	(Page 3)
	2b	NA. Clinical Trials Registry of India is a listed registry under WHO
Protocol version	3	
Funding	4	Funding Statement on page 16
Roles and responsibilities	5a	Authors Contribution on page 15
	5b	Page 3 under Trial registration section
	5c	Funding Statement on page 16
	5d	This is a process evaluation protocol so these do not apply in general but the whole evaluation will be overseen by the research team led by the PI
**Introduction**		
Background and rationale	6a	Description of research question and justification for undertaking the trial, including summary of relevant studies (published and unpublished) examining benefits and harms for each intervention Page 4-5
	6b	Explanation for choice of comparators Page 10
Objectives	7	Specific objectives or hypotheses Page 5 under Aims
Trial design	8	Description of trial design including type of trial (eg, parallel group, crossover, factorial, single group), allocation ratio, and framework (eg, superiority, equivalence, noninferiority, exploratory) Page 6 under study design
**Methods: Participants, interventions, and outcomes**		
Study setting	9	Description of study settings (eg, community clinic, academic hospital) and list of countries where data will be collected. Reference to where list of study sites can be obtained Page 6 under Study Setting

For peer review only

For peer review only - http://bmjopen.bmj.com/site/about/guidelines.xhtml

Eligibility criteria	10	Inclusion and exclusion criteria for participants. If applicable, eligibility criteria for study centres and individuals who will perform the interventions (eg, surgeons, psychotherapists) NA
Interventions	11a	Interventions for each group with sufficient detail to allow replication, including how and when they will be administered NA
	11b	Criteria for discontinuing or modifying allocated interventions for a given trial participant (eg, drug dose change in response to harms, participant request, or improving/worsening disease) NA
	11c	Strategies to improve adherence to intervention protocols, and any procedures for monitoring adherence (eg, drug tablet return, laboratory tests) NA
	11d	Relevant concomitant care and interventions that are permitted or prohibited during the trial NA
Outcomes	12	Primary, secondary, and other outcomes, including the specific measurement variable (eg, systolic blood pressure), analysis metric (eg, change from baseline, final value, time to event), method of aggregation (eg, median, proportion), and time point for each outcome. Explanation of the clinical relevance of chosen efficacy and harm outcomes is strongly recommended Pages 6 – 9 under Intervention Description
Participant timeline	13	Time schedule of enrolment, interventions (including any run-ins and washouts), assessments, and visits for participants. A schematic diagram is highly recommended (see Figure) NA
Sample size	14	Estimated number of participants needed to achieve study objectives and how it was determined, including clinical and statistical assumptions supporting any sample size calculations Table 1 on page 9
Recruitment	15	Strategies for achieving adequate participant enrolment to reach target sample size NA

Methods: Assignment of interventions (for controlled trials)

Allocation:

Sequence generation	16a	Method of generating the allocation sequence (eg, computer-generated random numbers), and list of any factors for stratification. To reduce predictability of a random sequence, details of any planned restriction (eg, blocking) should be provided in a separate document that is unavailable to those who enrol participants or assign interventions NA

Allocation concealment mechanism	16b	Mechanism of implementing the allocation sequence (eg, central telephone; sequentially numbered, opaque, sealed envelopes), describing any steps to conceal the sequence until interventions are assigned NA
Implementation	16c	Who will generate the allocation sequence, who will enrol participants, and who will assign participants to interventions NA
Blinding (masking)	17a	Who will be blinded after assignment to interventions (eg, trial participants, care providers, outcome assessors, data analysts), and how NA
	17b	If blinded, circumstances under which unblinding is permissible, and procedure for revealing a participant's allocated intervention during the trial NA
**Methods: Data collection, management, and analysis**		
Data collection methods	18a	Plans for assessment and collection of outcome, baseline, and other trial data, including any related processes to promote data quality (eg, duplicate measurements, training of assessors) and a description of study instruments (eg, questionnaires, laboratory tests) along with their reliability and validity, if known. Reference to where data collection forms can be found, if not in the protocol Table 2 on page 10
	18b	Plans to promote participant retention and complete follow-up, including list of any outcome data to be collected for participants who discontinue or deviate from intervention protocols NA
Data management	19	Plans for data entry, coding, security, and storage, including any related processes to promote data quality (eg, double data entry; range checks for data values). Reference to where details of data management procedures can be found, if not in the protocol NA
Statistical methods	20a	Statistical methods for analysing primary and secondary outcomes. Reference to where other details of the statistical analysis plan can be found, if not in the protocol NA
	20b	Methods for any additional analyses (eg, subgroup and adjusted analyses) NA
	20c	Definition of analysis population relating to protocol non-adherence (eg, as randomised analysis), and any statistical methods to handle missing data (eg, multiple imputation) NA
**Methods: Monitoring**		

Data monitoring	21a	Composition of data monitoring committee (DMC); summary of its role and reporting structure; statement of whether it is independent from the sponsor and competing interests; and reference to where further details about its charter can be found, if not in the protocol. Alternatively, an explanation of why a DMC is not needed NA
	21b	Description of any interim analyses and stopping guidelines, including who will have access to these interim results and make the final decision to terminate the trial NA
Harms	22	Plans for collecting, assessing, reporting, and managing solicited and spontaneously reported adverse events and other unintended effects of trial interventions or trial conduct NA
Auditing	23	Frequency and procedures for auditing trial conduct, if any, and whether the process will be independent from investigators and the sponsor NA
**Ethics and dissemination**		
Research ethics approval	24	Plans for seeking research ethics committee/institutional review board (REC/IRB) approval On page 3 and page 11 under Ethics and dissemination
Protocol amendments	25	Plans for communicating important protocol modifications (eg, changes to eligibility criteria, outcomes, analyses) to relevant parties (eg, investigators, REC/IRBs, trial participants, trial registries, journals, regulators) NA
Consent or assent	26a	Who will obtain informed consent or assent from potential trial participants or authorised surrogates, and how (see Item 32) Page 9 under data collection
	26b	Additional consent provisions for collection and use of participant data and biological specimens in ancillary studies, if applicable NA
Confidentiality	27	How personal information about potential and enrolled participants will be collected, shared, and maintained in order to protect confidentiality before, during, and after the trial Page 13, under Ethics and Dissemination
Declaration of interests	28	Financial and other competing interests for principal investigators for the overall trial and each study site Page 17, under Competing interests statement
Access to data	29	Statement of who will have access to the final trial dataset, and disclosure of contractual agreements that limit such access for investigators Page 13, under Ethics and Dissemination
Ancillary and post-trial care	30	Provisions, if any, for ancillary and post-trial care, and for compensation to those who suffer harm from trial participation. NA

For peer review only

Dissemination policy	31a	Plans for investigators and sponsor to communicate trial results to participants, healthcare professionals, the public, and other relevant groups (eg, via publication, reporting in results databases, or other data sharing arrangements), including any publication restrictions. On page 12 under Ethics and dissemination
	31b	Authorship eligibility guidelines and any intended use of professional writers NA
	31c	Plans, if any, for granting public access to the full protocol, participant-level dataset, and statistical code The full study protocol has already been published and is mentioned in the reference list (no. 22). Accessibility to the process evaluation data has been detailed on page 12 under Ethics and dissemination
**Appendices**		
Informed consent materials	32	Model consent form and other related documentation given to participants and authorised surrogates PISCF shared as an attachment on the online portal .
Biological specimens	33	Plans for collection, laboratory evaluation, and storage of biological specimens for genetic or molecular analysis in the current trial and for future use in ancillary studies, if applicable NA

*It is strongly recommended that this checklist be read in conjunction with the SPIRIT 2013 Explanation & Elaboration for important clarification on the items. Amendments to the protocol should be tracked and dated. The SPIRIT checklist is copyrighted by the SPIRIT Group under the Creative Commons "Attribution-NonCommercial-NoDerivs 3.0 Unported" license.

For peer review only

BMJ Open

Protocol for process evaluation of ARTEMIS cluster randomized controlled trial: an intervention for management of depression and suicide among adolescents living in slums in India

Journal:	*BMJ Open*
Manuscript ID	bmjopen-2023-081844.R1
Article Type:	Protocol
Date Submitted by the Author:	22-Mar-2024

Complete List of Authors:	Mukherjee, Ankita; The George Institute for Global Health India,<br>Yatirajula, Sandhya Kanaka; The George Institute for Global Health India, Research<br>Kallakuri, Sudha; The George Institute for Global Health<br>Paslawar, Srilatha; The George Institute for Global Health India, Research and Development<br>Lempp, Heidi; King's College London, Inflammation Biology<br>Raman, Usha; University of Hyderabad, Hyderabad, India, Department of Communication,<br>Essue, Beverley; University of Toronto, Institute of Health Policy, Management and Evaluation<br>Sagar, Rajesh; All India Institute of Medical Sciences, Department of Psychiatry<br>Singh, Renu; Young Lives, India<br>Peiris, David; The George Institute for Global Health; University of New South Wales<br>Norton, Robyn; The George Institute for Global Health; Imperial College London<br>Thornicroft, Graham; Institute of Psychiatry, Psychology and Neuroscience, King's College London, Centre for Global Mental Health and Centre for Implementation Science<br>Maulik, Pallab; The George Institute for Global Health; University of New South Wales Medicine & Health

**Primary Subject Heading**:	Mental health
Secondary Subject Heading:	Mental health
Keywords:	Adolescents < Adolescent, Protocols & guidelines < HEALTH SERVICES ADMINISTRATION & MANAGEMENT, Implementation Science, MENTAL HEALTH, Clinical Trial, Depression & mood disorders < PSYCHIATRY

SCHOLARONE™
Manuscripts

Protocol for process evaluation of ARTEMIS cluster randomized controlled trial: an intervention for management of depression and suicide among adolescents living in slums in India

Authors

1. Ankita Mukherjee (AM), The George Institute for Global Health, New Delhi, India, Email - AMukherjee@georgeinstitute.org.in
2. Sandhya Kanaka Yatirajula (SKY), Corresponding author), The George Institute for Global Health, New Delhi, India, Email – syatirajula@georgeinstitute.org.in
3. Sudha Kallakuri (SK), The George Institute for Global Health, Hyderabad, India, Email - skallakuri1@georgeinstitute.org.in
4. Srilatha Paslawar (SP), The George Institute for Global Health, Hyderabad, India, Email - SPaslawar@georgeinstitute.org.in
5. Heidi Lempp (HL), Centre for Rheumatic Diseases, Department of Inflammation Biology, School of Immunology and Microbial Sciences, Faculty of Life Sciences and Medicine, King's College London, UK, Email - heidi.lempp@kcl.ac.uk
6. Usha Raman (UR), Department of Communication, University of Hyderabad, Hyderabad, India, Email - usharaman@uohyd.ac.in
7. Beverley M. Essue (BE), Institute for Health Policy, Management and Evaluation, University of Toronto, Toronto, Canada, Email - beverley.essue@utoronto.ca
8. Rajesh Sagar (RS), Department of Psychiatry, All India Institute of Medical Sciences, New Delhi, Email - rsagar29@gmail.com
9. Renu Singh (ReS), Young Lives India, New Delhi, Email - renusab@gmail.com
10. David Peiris (DP), The George Institute for Global Health, UNSW Sydney, Sydney Australia, Email - dpeiris@georgeinstitute.org
11. Robyn Norton (RS), The George Institute for Global Health, UNSW Sydney, Sydney, Australia Imperial College, London, Email - rnorton@georgeinstitute.org
12. Graham Thornicroft (GT), Centre for Global Mental Health and Centre for Implementation Science, Institute of Psychiatry, Psychology and Neuroscience, King's College London, UK, Email - graham.thornicroft@kcl.ac.uk
13. Pallab K Maulik (PKM), The George Institute for Global Health, New Delhi, India, University of New South Wales, Sydney, Australia, Department of Brain Sciences, Imperial College London, London, United Kingdom, George Institute for Global Health, Imperial College London, United

For peer review only - http://bmjopen.bmj.com/site/about/guidelines.xhtml

For peer review only

Kingdom, Prasanna School of Public Health, Manipal Academy of Higher Education, Manipal, India, Email - pmaulik@georgeinstitute.org.in

For peer review only

For peer review only - http://bmjopen.bmj.com/site/about/guidelines.xhtml

Abstract

Introduction

There are around 250 million adolescents (10-19 years) in India. The prevalence of mental health related morbidity among adolescents in India is approximately 7.3%. Vulnerable sub-populations among adolescents such as those living in slum communities, are particularly at risk due to poor living conditions, financial difficulty, and limited access to support services The Adolescents' Resilience and Treatment nEeds for Mental Health in Indian Slums (ARTEMIS) is a cluster randomized controlled trial (cRCT) of an intervention intends to improve mental health of adolescents living in slum communities in India. The aim of this paper is to describe the process evaluation protocol for ARTEMIS trial. The process evaluation will help to explain the intervention outcomes and understand how and why intervention worked or did not work. It will identify contextual factors, intervention barriers and facilitators and the adaptations required for optimising implementation.

Methods

Case study method will be used and the data will include a mix of quantitative metrics and qualitative data. The UK Medical Research Council's guidance on evaluating complex interventions, the RE-AIM Framework and the APEASE criteria will be used to develop a conceptual framework and apriori codes for qualitative data analysis. Quantitative data will be analysed using descriptive statistics. Implementation fidelity will also be measured.

Discussion

The process evaluation will provide an understanding of outcomes and causal mechanisms that influenced any change in trial outcomes.

Ethics and Dissemination

Ethics Committee of the George Institute for Global Health India (Project number 17/2020) and the Research Governance and Integrity Team, Imperial College, London (ICREC Reference number: 22IC7718) have provided ethics approval. The Health Ministry's Screening Committee, has approved to the study (ID 2020-9770).

Trial Registration

The trial is registered with Clinical Trial Registry of India (Reference Number CTRI/2022/02/040307). The George Institute for Global Health, India is the trial sponsor.

Article Summary:

For peer review only - http://bmjopen.bmj.com/site/about/guidelines.xhtml

Strengths and limitations of this study

- This study will use existing implementation science theories and frameworks and qualitative and quantitative data will be triangulated to arrive at a comprehensive understanding of the intervention.
- This study will be guided by the MRC framework to developing and evaluating complex interventions and the case study method will also be used.
- Cases will be purposively selected based taking a maximum variation approach and both intervention and control sites will be selected as cases to the enable comparison, to understand contextual factors and to avoid the Hawthorne effect.

Key Words: process evaluation, protocol, Child and Adolescent Mental Health (CAMH), Low-middle income country (LMIC), cluster Randomized Control Trial (cRCT), depression, self-harm, anti-stigma, mHealth, electronic decision support system (EDSS), urban slum, India

Word count: 3459

For peer review only

For peer review only - http://bmjopen.bmj.com/site/about/guidelines.xhtml

INTRODUCTION

India has about 250 million adolescents aged 10-19 years, comprising nearly one-fifth of India's population [1]. The burden of mental health problems in adolescents is a growing concern globally[2]. In India, the prevalence of psychiatric disorders among adolescents is estimated to be about 7.3% [3]. Self-harm and depressive disorders are the leading cause of death and disability in the age group 15-19 years [4].

Mental health interventions for adolescents are important as 50% of adult mental disorders have an onset before age of 14 years [5]. Marginalized populations such as adolescents living in slum communities, are particularly vulnerable because of additional stressors related to poor living conditions, financial stress, and poor access to support services [6,7].

There has been limited research to test community-based mental health interventions for adolescents living in slums in India. A scoping review of mental health interventions among adolescents in India found that of the 11 interventions included in the review; nine were school-based, one community-based, and one was digital [8]). Most of the school-based programs used a life skills curriculum that resulted in improvements in depressive symptoms and overall mental well-being. The review recommended the need for more interventions for early and out-of-school adolescents to ensure that the most vulnerable adolescents were not missed out. An intervention to build mental health and resilience delivered by community-based peers among highly disadvantaged young women living in urban slums in Dehradun a city in north India, showed sustained improvements in anxiety and depression and attitudes to gender equality among study participants [9]. A randomized controlled trial of a 5-month resilience-based program among rural adolescent girls through government schools in the state of Bihar, India, delivered by local women showed that girls receiving the intervention (vs. controls) had better emotional resilience, self-efficacy, social-emotional assets, psychological wellbeing, and social wellbeing [10]. (Another school-based pilot study of POD (**P**roblems, **O**ptions, **D**o it!) Adventures (a smart phone based blended problem-solving game-based intervention for adolescents with or at risk of anxiety, depression and conduct difficulties) was helpful in managing their problems and stress and improving the mental health of 13 to 19 year olds enrolled in secondary schools in the Indian state of Goa [11]. The Adolescents' Resilience and Treatment nEeds for Mental Health in Indian Slums (ARTEMIS) cluster randomized control trial (cRCT) is testing a community-based intervention to improve mental health outcomes for adolescents living in urban slum clusters in India.

This paper presents the protocol for process evaluation of the ARTEMIS cRCT. ARTEMIS is a community-based cluster randomised control trial that aims to reduce depression and risk of suicide among adolescents living in slums. The intervention will use a mental health stigma reduction campaign with adolescents and parents of the study cohort to improve attitudes towards mental

For peer review only - http://bmjopen.bmj.com/site/about/guidelines.xhtml

health and improve help seeking. A technology-enabled strategy will be employed for screening, clinical diagnosis, and management of mental health problems (depression, other significant emotional or medically unexplained complaints and suicide risk) among adolescents by primary care doctors and community based non physician health workers (NPHWs) (described in detail below).

Process evaluations provide critical inputs in understanding how interventions work in particular contexts and thus, support implementation planning beyond the trial setting [12].

Aims

The aims of the process evaluation are to:

1. Assess implementation fidelity and understand how the intervention was implemented

2. Identify key contextual factors that impact intervention delivery and outcomes

3. Understand perceptions of different stakeholders about effectiveness, acceptability, and scalability of intervention components

4. Identify key facilitators and barriers in implementation of the intervention, and

5. Explain any adaptations to the intervention or intervention refinement during the study and their possible impact on the outcomes

METHODS

Conceptual Framework

The process evaluation will be guided by the MRC Framework for developing and evaluating complex interventions [13, 14]. The framework highlights the importance of examining the *implementation*, the *mechanism of impact* and their interaction with *contextual factors* to better understand how and why an intervention does or does not work. A recent update to the framework recognises that complex interventions have several phases including intervention development, feasibility assessment, implementation and evaluation, which may not always be sequential [15]. It recommends six core areas of inquiry at each phase before moving on to the next phase. They include (1) the intervention and its interaction with context; (2) the programme theory; (3) ways of engaging with diverse stakeholders; (4) ways of intervention refinement; (5) identifying key uncertainties; and (6) economic considerations. The process evaluation will focus on the first five areas of inquiry, and the overall objectives have been framed to address these key areas.

Study Setting

For peer review only

The ARTEMIS cRCT will be implemented in 60 slum clusters across two cities New Delhi and Vijayawada in India. In each city 30 slum clusters will be included. For this study, a slum cluster is defined as slums within wards or geographical areas identified as slums/resettlement colonies. A ward is a local authority area, typically used for electoral purposes. In certain cities of India, such as Mumbai and New Delhi, a ward is an administrative unit of the city region; a city area is divided into zones, which in turn contains numerous wards. New Delhi is a metropolis and one of the largest cities in India with a population of about 17 million and an estimated slum population of about 2 million [16,17]. Vijayawada is one of the largest cities in the state of Andhra Pradesh with an urban population of over 1.0 million[18,19] and an estimated slum population is estimated to be about 0.5 million[17]. The most widely spoken language in Delhi is Hindi, while in Vijayawada it is Telugu.

Patient and Public Involvement

Adolescent Expert Advisory Groups (AEAG) have been formed in each site. This group was involved throughout the intervention development phase in providing inputs and suggestions. Their contributions were critical for the co-creation of the anti-stigma content for adolescents. During the formative phase 34 meetings were held where the AEAG provided valuable feedback on ways to engage adolescents in the anti-stigma campaign.

Study Design

The study will use a mixed method, multiple case study design. Each slum cluster will constitute a 'case' for the study. A total of six cases or clusters will be purposively selected taking a maximum variation approach. Slums will be purposively selected to represent different contexts, coverage and reach of intervention as well as the ease or difficulty of implementing the intervention.

Intervention Description

The ARTEMIS cRCT will test an intervention to address depression, increased risk of self-harm/suicide, or other significant emotional or medically unexplained complaints among adolescents living in urban slums in two cities in India. ARTEMIS has two components. The first is a campaign that aims to reduce stigma related to mental health and improve attitudes and behaviours towards adolescents with depression or at increased risk of self-harm/suicide. The second is a technology-enabled mHealth platform with an integrated electronic decision support system (EDSS), to help primary care doctors and NPHWs to diagnose and treat adolescents at high-risk of depression, self-harm, or suicide.

Before randomization and before the start of the intervention, a team of trained field investigators will screen adolescents at high-risk of depression or suicide using Patient Health Questionnaire- 9 (PHQ-9), which is a standardised psychometric tool for screening depression and suicide risk in the

For peer review only - http://bmjopen.bmj.com/site/about/guidelines.xhtml

community [20, 21]. Adolescents who obtain a PHQ 9 score of ≥ 10 and/or a score of ≥2 to the suicide risk question on the PHQ-9 will be deemed as 'high-risk'. Due to the time delay between screening and randomisation and potential natural remission, a second screening will be carried out for adolescents identified at 'high-risk' before the baseline is administered. This process will help identify the final list of adolescents at 'high-risk' in all the clusters. The following information will be collected from the study cohort by the trained field investigators: socio-demographic characteristics, history of mental illness, treatment history, co-morbid conditions, stressful events experienced in the previous year, resilience, knowledge attitude and behaviours related to mental health and stigma associated with help seeking. A detailed protocol of the trial has been published [22].

A theory of change model for the intervention was developed with key stakeholders during the formative phase of the study in 2021. The logic model for intervention has been provided (Figure 1)

(Figure 1. Logic model about here)

Intervention Arm

In the intervention arm, the two components (i) the anti-stigma campaign and (ii) the technology-enabled mHealth based EDSS for screening, diagnosis and management of depression and self-harm will be delivered. (Figure 1).

Anti-stigma campaign

The anti-stigma campaign will include several Information Education and Communication (IEC) materials containing key messages around addressing mental health stigma, myths related to mental health, highlighting major stressors and their impact on mental health of adolescents and the importance of help seeking for mental health problems. The IEC materials will include posters, pamphlets, brochures, videos of persons with lived experience (PWLE), animation videos, audio dramas, games, magic shows, rallies, and street plays. The materials will be co-created with adolescents from the study sites. Adolescent Expert Advisory Groups (AEAGs) will be created in both locations consisting of adolescents from the community, with the purpose of involving them on working with the implementation team to co-create the anti-stigma campaign. Details of the process of forming AEAGs and their engagement in shaping the anti-stigma campaign has been published elsewhere [23].

Peer-educators

Based on findings from the formative phase of the study, peer groups were established comprising of adolescents from the community in the age group 13-19 years, some of whom were from the study

For peer review only - http://bmjopen.bmj.com/site/about/guidelines.xhtml

cohort. Their role will be to provide support to adolescents who approached them to discuss problems and to promote mental health awareness in the community. All the members of the peer groups will be provided basic training by the research team on mental health, safe practices while using social media, addiction and substance use and promoting mental wellbeing through engaging in pleasurable activities such a listening to music, sharing problems with trusted individuals, having friends, exercising, and eating healthily.

Technology-enabled EDSS

The EDSS platform has been developed to assist primary care doctors and NPHWs to screen, diagnose and manage depression, other significant emotional or medically unexplained complaints and suicide risk among adolescents. High-risk adolescents will be referred to the doctor electronically via the mHealth platform by the NPHWs. In each cluster, one doctor and several NPHWs will be trained. The NPHWs will be trained to use the EDSS to provide basic supportive advice to adolescents at high-risk of depression or self-harm, refer them to the primary care doctors, and track treatment advise given by the doctors and will follow-up for treatment adherence. The NPHWs will make regular home visits to encourage adolescents at high risk and their families to seek help from the doctor. The NPHWs will also use the EDSS to clinically monitor the severity of high-risk individuals at four months and at ten months after the start of the intervention. High-risk individuals will be provided referral cards to take to the primary care doctors. The primary care doctors will be trained to use an EDSS to clinically diagnose and manage adolescents with depression, other significant emotional or medically unexplained complaints and self-harm/suicide ideation based on WHO's mhGAP-Intervention Guide algorithm[24]. Adolescents needing specialist care will be referred to a psychiatrist in a government facility. The EDSS will also capture and store (on a cloud server) information regarding adolescents' visits and type of care provided by the primary care doctor-counselling/pharmacotherapy/combination of both/referral/follow-up. All data will be encrypted and protected using user-defined passwords. The information will be visible to the NPHWs, the project supervisors and research team only. A colour-coded traffic light system integrated in the application will assist the NPHWs to identify high-risk adolescents who need to be prioritised for followed up. The EDSS will also provide them with simple questions tailored to the priority level as indicated by the traffic-light coding system, that they can ask the adolescents during follow-up to ensure appropriate treatment adherence or follow-up with doctors or mental health specialists.

Control Arm

The control arm will receive enhanced usual care that will include general psychoeducation through pamphlets to raise awareness on common mental disorder in adolescents amongst the wider

For peer review only - http://bmjopen.bmj.com/site/about/guidelines.xhtml

community. Parents/guardians of adolescents identified at high-risk will be advised to consult a primary care doctor or mental health professional. Those with high scores on PHQ-9 (a score of >=15 on PHQ9) and/or at high risk of suicide (suicide score >=2), indicating severe depression and/or high risk of suicide will be asked to seek care immediately and will be given a list of health facilities (with contact details) where such treatment is available. All NPHWs will be asked to follow up with the adolescents and their families more intensively and will recommend that they visit a doctor/psychiatrist immediately.

Data Collection

Quantitative data for the process evaluation will draw on metrics collected sourced from the open-source medical record system (OpenMRS; (OpenMRS.org). The OpenMRS, is a standardized community-driven open-source software for storing and processing medical record information. Quantitative data will be collected throughout the intervention via the mHealth platform. This will include data on screening outcome, treatment seeking, follow ups, and treatment provided for the high-risk cohort. Data will also be used to understand usage patterns of the EDSS by primary care doctors and NPHWs. The mHealth platform will capture this data in tablets used by NPHWs and primary care doctors for screening, treatment, and follow-up of the high-risk cohort. This data will be utilised to assess reach, effectiveness, and service utilisation. Data on competency and fidelity measures will also be captured throughout the intervention.

Qualitative data collection will include key informant interviews (KII) and focus group discussions (FGDs) with various stakeholders including high-risk and non-high-risk cohort in both arms, parents, primary care doctors, NPHWs, members of the AEAG and peer leaders. A total of 33 FGDs (each with 6-12 participants) and 28 interviews are planned across the six purposively selected slum clusters equally divided between both sites. This will include four intervention clusters (two each in Delhi and Vijayawada) and 2 control clusters (one each in Delhi Vijayawada) (Table 1). The KIIs and FGDs with stakeholders will help capture information related to fidelity of the intervention and identify any gaps that could be addressed. Written informed consent will be sought from all participants selected for KIIs and FGDs.

(Table 1 about here)

Data Analysis

The conceptual framework for the study and how it integrates with the key parameters from the MRC frameworks is provided in Table 2.

(Table 2 about here)

For peer review only - http://bmjopen.bmj.com/site/about/guidelines.xhtml

The case study methodology will also be used as it is recognised to be suitable to study complex interventions in different contexts and helps to capture 'the complexity of the case, the relationship between the intervention and the context and how the intervention worked (or did not)' ([25]. Following Pfadenhauer et al (2016) we define context as 'a set of characteristics and circumstances that consist of active and unique factors that surround the implementation. As such it is not a backdrop for implementation but interacts, influences, modifies and facilitates or constrains the intervention and its implementation' [26]. We will be selecting both intervention and control sites as cases to avoid the Hawthorne effect. Two slum cluster in the control arm will also be selected for the case study to enable comparison, to understand contextual factors and any changes to usual care in the absence of an intervention. The importance of studying changes in control arms has been recognised as being useful in understanding intervention impact [27]. A multiple case study design will be use where each case will be analysed at the case level, but we will also pull together all the cases from the intervention and control arms for a more in-depth analysis and comparison. Basic descriptive analysis will be conducted with the quantitative data. Qualitative data will be transcribed by a professional vendor and the transcripts will be read by at least two members of the research team and coded. Coding will be carried out using a priori codes based on the conceptual framework (Table 3) with the help of N-Vivo 12 software.

(Table 3 about here)

Any additional codes emerging during the analysis will be added to the coding framework. Members of the research team will read transcripts separately and come up with codes. The research team will then compare and combine their codes to evaluate their fit and usefulness and will examine the differences in code to see if any new insights can be generated. The code list will be finalised after a discussion between the researchers to establish agreement among coders. Qualitative and quantitative data will be triangulated to arrive at a comprehensive understanding of the intervention. Triangulation helps in confirming the findings from quantitative and qualitative data thereby increasing validity as well as leading to a better understanding of phenomena being studied [28]

Assessment of Implementation Fidelity

Implementation fidelity refers to the extent to which an intervention was implemented as intended. The RE-AIM [29] (Reach, Efficacy, Adoption, Implementation and Maintenance) framework will be applied to understand implementation outcomes. Additionally, the APEASE (Affordability, Practicability, Effectiveness and cost-effectiveness, Acceptability, Safety/Side Effects and , Equity) criteria for evaluation of behaviour change interventions [30] will be utilised [31,32]. The framework has some similarities with RE-AIM, but includes two additional parameters, i.e., practicability and

For peer review only - http://bmjopen.bmj.com/site/about/guidelines.xhtml

safety. An intervention is practicable if 'it can be delivered as designed through the means intended to the target population'[30].

We will assess fidelity in 3 components (a) delivery of anti-stigma campaign, (b) implementation of the mHealth component, including trainings. Indicators have been developed for all these components to measure frequency of exposure and coverage. The quality of training provided to primary doctors on the mhGAP, NPHWs on the priority listing app and peer educators on promoting mental wellbeing and competency will be assessed through various tools including a post training satisfaction survey, pre-post-test self-assessment checklists as well as though rating by trainers and staff on competency checklists.

Discussion

This paper describes the design of a mixed method process evaluation for the ARTEMIS cRCT, which involves an anti-stigma campaign and a mobile device-based decision support system for primary care doctors and non-physician health workers, to improve treatment of adolescents at high-risk of depression, other significant emotional or medically unexplained complaints and suicide risk.

The process evaluation will help us understand and explain key causal mechanisms that led to change. and will therefore strengthen the understanding of the implementation process by highlighting various barriers and facilitators. Furthermore, it will provide an understanding of how local context played a role in the way the intervention was implemented and help identify the need and impact of any adaptations made to the intervention. The process evaluation will provide stakeholder perspectives on aspects of the intervention that worked and those that need further adaptation.

Strengths and Limitations

The protocol was developed using existing implementation science theories and frameworks and combines qualitative and quantitative measures to understand key aspects of how the intervention was implemented and which aspects worked or need further improvement. The MRC framework, which is a comprehensive framework, widely used in the field of implementation science will be used. The case study method using maximum variation approach will be used to make suitable comparisons among different contexts and avoid Hawthorne. The ARTEMIS intervention does not directly address social determinants which impact mental health outcomes. In this study we will collect additional qualitative and quantitative data on social support, socioeconomic conditions, education, and the local context of participants which will help to better understand and explain linkages between mental health outcomes and social determinants.

Ethics and Dissemination

For peer review only - http://bmjopen.bmj.com/site/about/guidelines.xhtml

The study has received formal ethics approval from the Ethics Committee of the George Institute for Global Health India on 04 September 2020 (Project number 17/2020). The study also received formal ethics approval from the Research Governance and Integrity Team, Imperial College, London on 8th June 2022 (ICREC Reference number: 22IC7718). The Health Ministry's Screening Committee (HMSC), Indian Council for Medical Research (ICMR) has also provided approval to the project (ID 2020-9770). Findings will be disseminated to study participants and other stakeholders at a policy symposium. All identifiable personal data will be stored in password protected secured servers located at The George Institute for Global, India office in Hyderabad. Only deidentified data will be disseminated. Data will be available with the PI on an accessible data repository, which can be accessed by other researchers, subject to a formal request to the PI to access the data for research purposes.

Trial Status

At the time of writing the paper, the intervention had started in both sites. Randomization was executed in Vijayawada and in Delhi on 12th December 2022. Intervention components were implemented in both the sites.

References

1. UNICEF: On My Mind: The State of the World's Children 2021. New York: United Nations Children's Fund; 2021. Available from: https://www.unicef.org/media/114636/file/SOWC-2021-full-report-English.pdf
2. Bor W, Dean AJ, Najman J, Hayatbakhsh R: Are child and adolescent mental health problems increasing in the 21st century? A systematic review. The Australian and New Zealand journal of psychiatry. 2014.48(7):606–16. Available from: https://pubmed.ncbi.nlm.nih.gov/24829198/
3. Gururaj G, Varghese M, Benegal V, Rao GN, Pathak K, Singh LK, Mehta RY, Ram D, Shibukumar TM, Kokane A, Lenin Singh RK, Chavan BS, Sharma P, Ramasubramanian C, Dalal PK, Saha PK , Deuri SP, Giri AK, Kavishvar AB, Sinha VK, Thavody J, Chatterji R, Akoijam MR: National Health Survey of India 2015-16: Prevalence, patterns and outcomes. Bangalore; 2016.
4. Joshi R, Alim M, Maulik PK, Norton R: A contemporary picture of the burden of death and disability in Indian adolescents: data from the Global Burden of Disease Study. International Journal of Epidemiology. 2017.46(6):2036. Available from: /pmc/articles/PMC5837726/

For peer review only

5. Patton GC, Sawyer SM, Santelli JS, Ross DA, Afifi R, Allen NB, Arora M, Azzopardi P, Baldwin W, Bonell C, Kakuma R, Kennedy E, Mahon J, McGovern T, Mokdad AH, Patel V, Petroni S, Reavley N, Taiwo K, Waldfogel J, Wickremarathne D, Barroso C, Bhutta Z, Fatusi AO, Mattoo A, Diers J, Fang J, Ferguson J, Ssewamala F, Viner RM: Our future: a Lancet commission on adolescent health and wellbeing. Lancet (London, England). 2016.387(10036):2423. Available from: /pmc/articles/PMC5832967/

6. Culbreth R, Swahn MH, Ndetei D, Ametewee L, Kasirye R: Suicidal Ideation among Youth Living in the Slums of Kampala, Uganda. International Journal of Environmental Research and Public Health 2018, Vol. 15, Page 298. 2018.15(2):298. Available from: https://www.mdpi.com/1660-4601/15/2/298/htm

7. Subbaraman R, Nolan L, Shitole T, Sawant K, Shitole S, Sood K, Nanarkar M, Ghannam J, Betancourt TS, Bloom DE, Patil-Deshmukh A: The psychological toll of slum living in Mumbai, India: a mixedmethods study. Social science & medicine (1982). 2014.119:155. Available from: /pmc/articles/PMC4252879/

8. Mehra, D., Lakiang, T., Kathuria, N., Kumar, M., Mehra, S., & Sharma, S. (2022). Mental Health Interventions among Adolescents in India: A Scoping Review. *Healthcare (Basel, Switzerland)*, *10*(2), 337. https://doi.org/10.3390/healthcare10020337

9. Mathias, K., Pandey, A., Armstrong, G. *et al.* Outcomes of a brief mental health and resilience pilot intervention for young women in an urban slum in Dehradun, North India: a quasi-experimental study. *Int J Ment Health Syst* **12**, 47 (2018). https://doi.org/10.1186/s13033-018-0226-y

10. Leventhal, Katherine Sachs et al. “Building psychosocial assets and wellbeing among adolescent girls: A randomized controlled trial.” *Journal of adolescence* vol. 45 (2015): 284-95. doi:10.1016/j.adolescence.2015.09.011

11. Gonsalves, Pattie P et al. “App-based guided problem-solving intervention for adolescent mental health: a pilot cohort study in Indian schools.” *Evidence-based mental health* vol. 24,1 (2021): 11-18. doi:10.1136/ebmental-2020-300194

12. Lockwood I., Walker RM, Latimer S., Chaboyer W., Cooke M., Gillespie BM. Process evaluations undertaken alongside randomised controlled trials in the hospital setting: A scoping review. Contemporary Clinical Trials Communications, Volume 26, 2022, 100894,ISSN 2451-8654, https://doi.org/10.1016/j.conctc.2022.100894

For peer review only - http://bmjopen.bmj.com/site/about/guidelines.xhtml

13. Moore GF, Audrey S, Barker M, Bond L, Bonell C, Hardeman W, Moore L, O'Cathain A, Tinati T, Wight D, Baird J: Process evaluation of complex interventions: Medical Research Council guidance. BMJ (Online). 2015.350.

14. Craig P, Dieppe P, Macintyre S, Mitchie S, Nazareth I, Petticrew M: Developing and evaluating complex interventions: The new Medical Research Council guidance. BMJ. British Medical Journal Publishing Group; 2008. p. 979–83. Available from: www.mrc.ac.uk/

15. Skivington K, Matthews L, Simpson SA, Craig P, Baird J, Blazeby JM, Boyd KA, Craig N, French DP, McIntosh E, Petticrew M, Rycroft-Malone J, White M, Moore L: A new framework for developing and evaluating complex interventions: update of Medical Research Council guidance. BMJ. 2021.374. Available from: https://www.bmj.com/content/374/bmj.n2061

16. Office of the Registrar General & Census Commissioner India: Primary Census Abstract Data Tables. [accessed 2022 Jun 29]. Available from: https://web.archive.org/web/20190714165104/http://censusindia.gov.in/pca/pcadata/pca.html

17. Ministry of Housing and Urban Poverty Alleviation Government of India: Slums in India : A Statistical Compendium 2015. 2015 [accessed 2022 Jul 19]. Available from: http://nbo.nic.in/pdf/SLUMS_IN_INDIA_Slum_Compendium_2015_English.pdf

18. Government of Andhra Pradesh: Handbook of Statistics 2019: Krishna District. 2019.

19. Office of the Registrar General & Census Commissioner: Population Finder 2011. Ministry of Home Affairs,Government of India [Internet]. Available from: https://censusindia.gov.in/census.website/data/population-finder

20. Pfizer Inc.: Patient Health Questionnaire- 9 (PHQ-9). 2010 [accessed 2020 Jul 20]. Available from: https://www.phqscreeners.com/images/sites/g/files/g10060481/f/201412/PHQ9_English for India.pdf

21. Kroenke K, Spitzer RL, Williams JBW: The PHQ-9: Validity of a brief depression severity measure. Journal of General Internal Medicine. 2001.16(9):606–13. Available from: /pmc/articles/PMC1495268/?report=abstract

22. Yatirajula SK, Kallakuri S, Paslawar S, Mukherjee A, Bhattacharya A, Chatterjee S, Sagar R, Kumar A, Lempp H, Raman U, Singh R, Essue B, Billot L, Peiris D, Norton R, Thornicroft G, Maulik PK: An intervention to reduce stigma and improve management of depression, risk of

For peer review only - http://bmjopen.bmj.com/site/about/guidelines.xhtml

suicide/self-harm and other significant emotional or medically unexplained complaints among adolescents living in urban slums: protocol for the ARTEMIS project. Trials 2022 23:1. 2022.23(1):1–15. Available from: https://trialsjournal.biomedcentral.com/articles/10.1186/s13063-022-06539-8

23. Yatirajula SK, Kallakuri S, Paslawar S, Mukherjee A, Khan NA, Kumari K, Sagar R, Thornicroft G, Maulik PK: Engaging with adolescents to navigate the Adolescents' Resilience and Treatment nEeds for Mental health in Indian Slums (ARTEMIS) trial. Journal of Global Health.2022.12:03048

24. WHO: mhGAP Intervention Guide for mental, neurological and substance use disorders in non- specialized health settings (Version 1.0). Geneva; 2010 [accessed 2020 Jun 11]. Available from: www.who.int/mental_health/mhgap

25. Yin R. Case study research and applications: design and methods. Los Angeles: Sage Publications Inc; 2018

26. Pfadenhauer L, Rohwer A, Burns J, Booth A, Lysdahl KB, Hofmann B, Gerhardus A, Mozygemba K, Tummers M, Wahlster P, et al. Guidance for the assessment of context and implementation in health technology assessments (HTA) and systematic reviews of complex interventions: the Context and Implementation of Complex Interventions (CICI) framework: Integrate-HTA; 2016

27. Rachel Jewkes, Andrew Gibbs, Esnat Chirwa & Kristin Dunkle (2020) What can we learn from studying control arms of randomised VAW prevention intervention evaluations: reflections on expected measurement error, meaningful change and the utility of RCTs, Global Health Action, 13:1, DOI: 10.1080/16549716.2020.1748401

28. Bekhet Ak., Zauszniewski JA. Methodological triangulation: an approach to understanding data. Nurse Researcher. 20, 2, 40-43. doi**:** 10.7748/nr2012.11.20.2.40.c9442

29. Waitzkin H: Different Views A Marxian Interpretation of the Growth And Development of Coronary Care Technology. American Journal of Public Health. 1979.69(12):1260–8. Available from: http://ajph.aphapublications.org/doi/pdf/10.2105/AJPH.69.12.1260

30. Michie S, Atkins L, Gainforth HL: Changing Behaviour to Improve Clinical Practice and Policy. In: Gonçalves A, Azevedo A, Lobo F, editors. Novos Desafios, Novas Competências: Contributos Atuais da Psicologia. Braga,Portugal: Axioma - Publicações da Faculdade de Filosofia; 2016. p. 41–60.

For peer review only

For peer review only - http://bmjopen.bmj.com/site/about/guidelines.xhtml

31. Michie S, West R, Rogers MB, Bonell C, Rubin GJ, Amlôt R: Reducing SARS-CoV-2 transmission in the UK: A behavioural science approach to identifying options for increasing adherence to social distancing and shielding vulnerable people. British Journal of Health Psychology. 2020.25(4):945–56.

32. Brierley ML, Smith LR, Bailey DP, Ojo SO, Hewson DJ, Every SA, Staines TA, Chater AM: Evaluating a multi-component intervention to reduce and break up office workers' sitting with sit-stand desks using the APEASE criteria. BMC Public Health. 2022.22(1):1–15. Available from: https://bmcpublichealth.biomedcentral.com/articles/10.1186/s12889-022-12794-w

Author contribution statement

The paper was conceptualised by PKM. AM wrote the original draft with contributions from SKY. PKM commented on multiple drafts before sending a prefinal version to everyone listed as authors. SK, SP, HL, UR, BME, RS, ReS, DP, RN, GT reviewed the draft and provided critical intellectual inputs and comments to the draft. PKM leads the implementation of the trial in India along with SKY, SK, SP and AM. SKY, SK and SP played a key role in implementing research activities in the sites. All authors read and approved the final manuscript.

Funding

Adolescents' Resilience and Treatment nEeds for Mental health in Indian Slums (ARTEMIS) is funded by UK Research and Innovation/Medical Research Council (UKRI/MRC), Grant no: MR/S023224/1. SKY, SK, and SP are all partially or fully supported by ARTEMIS. PKM and AM are partially supported through NHMRC/GACD grant (SMART MentalHealth-APP1143911) and ARTEMIS. PKM is the PI for ARTEMIS and Co-PI for SMART Mental Health. HL and GT are partly support by ARTEMIS. GT is also supported by the National Institute for Health Research (NIHR) Applied Research Collaboration South London at King's College London. The views expressed are those of the author(s) and not necessarily those of the NHS, the NIHR or the Department of Health and Social Care. GT is also supported by the MRC-UKRI in relation to the Indigo Partnership (MR/R023697/1) awards. The funding agency had no role in the writing of the manuscript or the decision to submit it for publication.

Competing interests statement

We declare that the George Institute for Global Health has a part-owned social enterprise, George Health Enterprises, which has commercial relationships involving digital health innovations.

Acknowledgements

For peer review only - http://bmjopen.bmj.com/site/about/guidelines.xhtml

We acknowledge the contribution of all members of the Adolescent Expert Advisory Group in Delhi and Vijayawada who has generously contributed their time and expertise to the development of ARTEMIS. GT is supported by the National Institute for Health and Care Research (NIHR) Applied Research Collaboration South London (NIHR ARC South London) at King's College Hospital NHS Foundation Trust. The views expressed are those of the author(s) and not necessarily those of the NIHR or the Department of Health and Social Care.

Data Availability Statement

This is a process evaluation protocol paper; no data has been collected as yet and therefore there is no data that can be shared. However the data will be made available on reasonable request after publication of findings.

For peer review only

For peer review only - http://bmjopen.bmj.com/site/about/guidelines.xhtml

Table 1: Qualitative Data Collection Plan

**Intervention Arm**		
Data Sources	**FGDs Planned**	**Interviews planned**
Non-physician Health Workers (NPHWs)	4	-
Adolescents from high-risk cohort	8	-
Adolescents from nonhigh-risk cohort	4	
Parents of high-risk adolescents in the study cohort		24
Members of Adolescent Expert Advisory Groups (AEAG)	5	-
Peer Leaders	4	-
Field Staff	2	-
Key implementation team members	1	
Primary Care Doctors	-	4
**Control Arm**		
Non-Physician Health Workers (NPHWs)	2	-
Adolescents from study cohort	4	-
TOTAL	34	28

For peer review only - http://bmjopen.bmj.com/site/about/guidelines.xhtml

Table 2. Conceptual Framework for Process Evaluation

Broad Area of Enquiry	Domains of Inquiry	Key Questions/Process Measures	Data Source
CONTEXT	Differences in context	• What are the differences in terms of geography, slum conditions, socio-cultural norms, economic between the sites and among the clusters?<br>• What are the contextual factors which affect programme delivery, stakeholder response and intervention uptake in different settings?<br>• Who were the key stakeholders who could play a role in improving intervention outcomes?	Secondary data;<br>Formative research data<br>FGDs with project staff<br><br>FGDs with key members of the implementation team
	Intervention adaptions based on context	• What are some of the key contextual factors which influenced the overall implementation of the intervention?<br>• What were some of the context specific adaptations that were made to address emerging challenges?	FGDs with project staff<br><br>FGDs with key members of the implementation team<br><br>Project documentation completed during the project
	Contextual Barriers and Facilitators	• What are key facilitators in different contexts that helped in intervention delivery and uptake?	FGDs with project staff

For peer review only - http://bmjopen.bmj.com/site/about/guidelines.xhtml

		• What are the barriers in various contexts for implementing the intervention components?	FGDs with key members of the implementation team

**IMPLEMENTATION**	Implementation fidelity	Was the intervention delivered as it was planned?	Program records and documents; Observation and rating
	Intervention Reach	• What was the coverage of the different *anti-stigma campaign* methods, in terms of:<br>- Total persons reached (including age and gender-wise break-up)<br>- Slums and clusters covered.<br>- Number and proportion of high-risk cohort reached.<br>- Number and proportion of non-high-risk cohort reached.<br>- Key stakeholders reached.<br>• What was the reach of the mHealth services:	Project records and documents<br>Backend data<br>FGDs with project staff<br>FGDs with key members of the implementation team<br>Interviews with NPHWs

For peer review only - http://bmjopen.bmj.com/site/about/guidelines.xhtml

		- Number and proportion of high-risk cohort in the intervention arm provided counselling or follow-up services by NPHWs.<br>- Number and proportion of high-risk cohort from the intervention arm who sought care with the primary care doctor.<br>- Number and proportion high risk-cohort from the control arm who sought care.<br><br>• Did the NPHWs face any challenge in reaching out adolescents and their families during the intervention?	
	Intervention effectiveness	• What was the perception of adolescents and key stakeholders about the utility effectiveness of the anti-stigma campaign?<br>• What are some of the key take home messages from anti stigma campaign for adolescents and community members?<br>• What was the perception of NPHWs about impact of anti-stigma campaign in their community?	Community satisfaction survey done at the end of street play performance.<br><br>Outcome survey data;<br>Backend data<br><br>FGDs with adolescents<br><br>FGDs with AEAG and peer leaders

For peer review only - http://bmjopen.bmj.com/site/about/guidelines.xhtml

		• What is the association between exposure to anti stigma content with changes in KAB scores and care seeking?<br>• What is the perception of NPHWs about effectiveness of mHealth based EDSS for management of depression and self-harm in adolescents?<br>• What is the perception of doctors about effectiveness of mHealth based EDSS for community-based management of depression and self-harm in adolescents?	Interviews with parents
	Intervention acceptability and adoption	• What was the experiences of NPHWs in using EDSS for providing care (challenges, perceived benefits, potential for routine use of mHealth)?<br>• What was the experiences of doctors about using EDSS for providing care (challenges, perceived benefits, potential for routine use of mHealth)?<br>• What were some key features of use of EDSS by doctors:	Back-end data<br>FGDs with NPHWs<br>Interviews with doctors<br>Interview with high-risk adolescents and their parents

For peer review only - http://bmjopen.bmj.com/site/about/guidelines.xhtml

		- Average time taken for diagnosis and identification of treatment plan using mhGAP over time.<br>- Association between type of CMD and time taken for diagnosis and identification of treatment plan using mhGAP.<br><br>• What were the perceptions of high-risk adolescents and their parents about ease of getting treatment through mHealth?	
	Practicability	• What were the challenges/barriers in delivering of the different strategies of anti-stigma campaign to adolescents?<br>• What were the challenges of the electronic decision support systems by NPHWs and doctors for diagnosis and management of depression and self-harm among adolescents?<br>• Doctor's experiences of interactions with adolescents<br>• Experiences of NPHWs with adolescents during follow up and motivating them and their families to visit the doctor initially and for follow up	FGDs with NPHWs<br><br>Interviews with doctors<br><br>FGDs with project staff

For peer review only - http://bmjopen.bmj.com/site/about/guidelines.xhtml

	Safety and Side Effects	• Were there any unanticipated negative consequences because of the anti-stigma campaign?<br>• Were there any safety concerns related to using mHealth based EDSS platform for management of depression and self-harm among adolescents?	FGDs with adolescents<br>FGDs with NPHWs<br>Interviews with doctors<br>FGDs with project staff

**MECHANISM OF IMPACT**	Variation in outcomes	What kind of cluster level variation is observed in in the outcomes? What works, for whom and in what context?	Outcome data<br>Backend data;<br>FGDs with NPHWs<br>Interviews with doctors<br>FGDs with project staff
	Unexpected outcomes	What are some unexpected outcomes and what factors can be attributed to them?	Outcome data<br>Back-end data;<br>FGDs with NPHWs<br>Interviews with doctors<br>FGDs with project staff

For peer review only

	Theory of Change (ToC)/ Pathways to achieving outcomes	What were some of the key pathways to improved outcomes for high-risk adolescents?<br>What changes and adaptations were made to the ToC during the intervention?<br>What were some of the key assumptions regarding causal mechanisms that need to be modified?	Outcome data<br>Back-end data<br>Project documents<br>FGDs with project staff

For peer review only - http://bmjopen.bmj.com/site/about/guidelines.xhtml

Table 3: Qualitative Data Collection Areas of Inquiry

Type of Group/Individual	Some areas of inquiry
Non-Physician Health Workers	• Perceptions on effectiveness and appropriateness of training received.<br>• Experience of using mHealth platform for delivering the intervention including challenges or facilitators<br>• Perception about effectiveness anti-stigma campaign in improving help seeking.<br>• Facilitators and barriers in treatment seeking by high-risk cohort.<br>• Overall experience of participating in the trial.<br>• Perception about any personal benefits/harms due to their association with the project.<br>• Perception about what role they see for themselves in the community once the project comes to an end.<br>• Any spillover effects-experiences of helping or sharing information with other adolescents not in the study cohort.
Project Field staff in each site	• Barriers or facilitators in delivering anti-stigma campaign in the community.<br>• Barriers and facilitators in delivering mHealth component.<br>• Perceived factors which explain high/low treatment seeking in different clusters.<br>• Key lessons learnt and suggestions for implementation of intervention components.<br>• Perceptions on training appropriateness, effectiveness and methods.

For peer review only - http://bmjopen.bmj.com/site/about/guidelines.xhtml

Study participants from high-risk cohort in intervention arm	• Perception appropriateness of anti-stigma content and methods for adolescents.<br>• Perceptions about impact and effectiveness of anti-stigma campaign in community.<br>• Any reported change in perception about mental health related stigma and help seeking in self or family.<br>• Perception about case detection, treatment and follow-up through EDSS.<br>• Facilitators and barriers in help-seeking by adolescents<br>• Experience of care and perception about quality of care, provided by doctors.<br>• Positive/negative experiences as a study participant.<br>• Perception about benefits/effectiveness of the intervention.<br>• Any spillover effects-experience of helping or sharing information with other adolescents not in the study cohort.
Study participants from non-high-risk cohort in intervention arm	• Perceptions about impact and effectiveness of anti-stigma campaign in community.<br>• Any reported changes in perception about mental health related stigma and help seeking in self or family.<br>• Facilitators and barriers in treatment seeking.<br>• Positive/negative experiences as a study participant.<br>• Perception about benefits/effectiveness of the intervention.<br>• Any spillover effects-experience of helping or sharing information with other adolescents not in the study cohort.
Study participants from (high-risk and non-high-risk	• Perception about impact and effectiveness of anti-stigma content.<br>• Any reported change in perception about mental health related stigma and help seeking in self or family.<br>• Facilitators and barriers in help seeking.

For peer review only - http://bmjopen.bmj.com/site/about/guidelines.xhtml

cohort) in the control arm	• Experience as a study participant.
Parents of adolescents in the study cohort	• Perception about impact and effectiveness of anti-stigma content.<br>• Any reported change in perception about mental health related stigma and help seeking in self or family.<br>• Knowledge about key stressors and symptoms of mental health problems in adolescents.<br>• Facilitators and barriers in help-seeking by adolescents.<br>• Experience of help seeking.<br>• Perception about benefits/effectiveness of the intervention.
Adolescent expert Advisory Groups in each site	• Perception about their role on co-creation of anti-stigma material and feedback on the whether the anti-stigma material was able reflect their suggestions.<br>• Perception about impact and effectiveness of anti-stigma content among adolescents and community members.<br>• Any reported change in perception about mental health related stigma and help seeking in self or family.<br>• Perceived personal benefit/challenges in being an AEAG member.<br>• Perception about benefit and effectiveness of mHealth component.<br>• Facilitators and barriers in help seeking by high-risk adolescents.<br>• Suggestions on improving engagement/involvement of younger adolescents in AEAGs and the co-creation process.

For peer review only - http://bmjopen.bmj.com/site/about/guidelines.xhtml

Peer Group - Intervention arm	• Perceptions on effectiveness and appropriateness of training received.<br>• Perceptions about impact and effectiveness of anti-stigma campaign in community.<br>• Perception about effectiveness anti-stigma campaign in improving help seeking by adolescents.<br>• Facilitators and barriers in treatment seeking by high-risk cohort.<br>• Experience if any of providing help to adolescents in the community who reached out to the peer group.<br>• Overall experiences of participating in the trial.<br>• How does the learnings from the project/trainings will be useful beyond the project period.
Doctors from intervention arm	• Perceptions on effectiveness and appropriateness of training received.<br>• Experience of using technology-based decision support system for diagnosis and management of depression and self-harm among adolescents.<br>• Perceived effectiveness anti-stigma campaign.<br>• Possible facilitators and barriers to scaling up mHealth component.<br>• Overall experience of participating in the trial including any challenges.<br>• Facilitators and barriers in help seeking and treatment adherence among adolescents.<br>• Whether learnings from the project/trainings will be useful beyond the project period.
Study participants from high-risk cohort in intervention arm who visited the	• Experience of seeking care from primary care doctor and perception about quality of care.<br>• Level of comfort/discomfort in sharing their mental distress experiences with doctors.<br>• Perceived benefit, if any, as a result of treatment.<br>• Barriers and facilitators for seeking treatment from a specialist (in case of referral).<br>• Views about help-seeking in future or continuing care if advised by the doctor.

For peer review only

doctor and were given medical and/or psychological treatment.	• Positive/negative experiences as a study participant.<br>• Suggestions for the programme in the future.
Parents/guardians of study participants from high-risk cohort in intervention arm who visited the doctor	• Perception about impact and effectiveness of anti-stigma content.<br>• Any reported change in perception about mental health related stigma and help seeking in self or family.<br>• Knowledge about key stressors and symptoms of mental health problems in adolescents.<br>• Knowledge perception about mental health problems of their ward.<br>• Barriers and facilitators for seeking mental healthcare.<br>• Experience of seeking mental health care for their wards.<br>• Perceived benefit, if any, as a result of treatment.<br>• Views about help-seeking in future or continuing mental health care if advised by the doctor.<br>• Suggestions for the programme in the future.

For peer review only

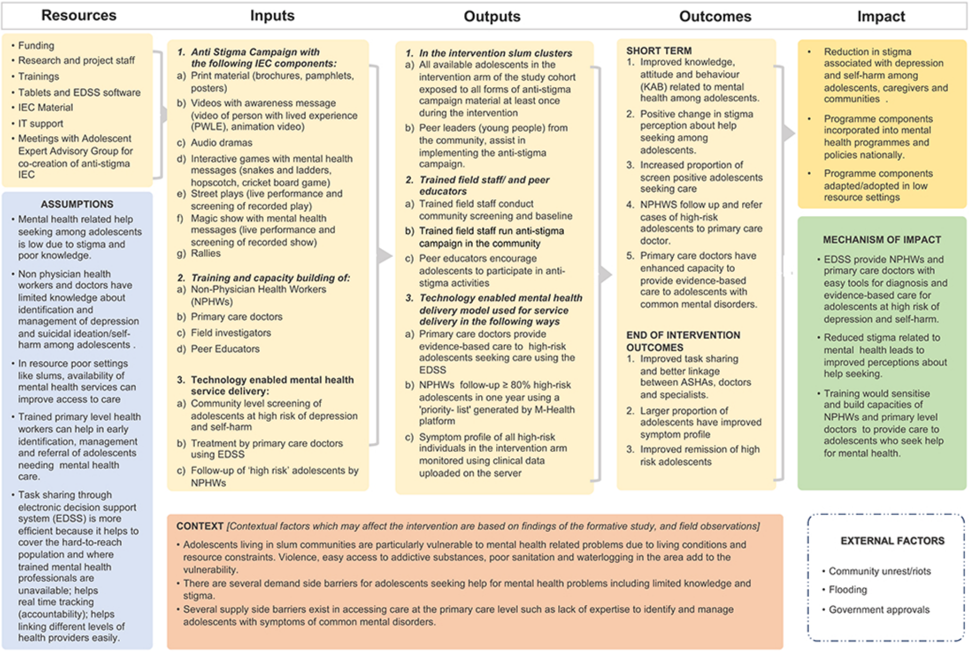

90x60mm (300 x 300 DPI)

SPIRIT

STANDARD PROTOCOL ITEMS: RECOMMENDATIONS FOR INTERVENTIONAL TRIALS

SPIRIT 2013 Checklist: Recommended items to address in a clinical trial protocol and related documents*

Section/item	Item No	Description
**Administrative information**		
Title	1	(Page 1)
Trial registration	2a	(Page 3)
	2b	NA. Clinical Trials Registry of India is a listed registry under WHO
Protocol version	3	
Funding	4	Funding Statement on page 16
Roles and responsibilities	5a	Authors Contribution on page 15
	5b	Page 3 under Trial registration section
	5c	Funding Statement on page 16
	5d	This is a process evaluation protocol so these do not apply in general but the whole evaluation will be overseen by the research team led by the PI
**Introduction**		
Background and rationale	6a	Description of research question and justification for undertaking the trial, including summary of relevant studies (published and unpublished) examining benefits and harms for each intervention Page 4-5
	6b	Explanation for choice of comparators Page 10
Objectives	7	Specific objectives or hypotheses Page 5 under Aims
Trial design	8	Description of trial design including type of trial (eg, parallel group, crossover, factorial, single group), allocation ratio, and framework (eg, superiority, equivalence, noninferiority, exploratory) Page 6 under study design
**Methods: Participants, interventions, and outcomes**		
Study setting	9	Description of study settings (eg, community clinic, academic hospital) and list of countries where data will be collected. Reference to where list of study sites can be obtained Page 6 under Study Setting

For peer review only

Eligibility criteria	10	Inclusion and exclusion criteria for participants. If applicable, eligibility criteria for study centres and individuals who will perform the interventions (eg, surgeons, psychotherapists) NA
Interventions	11a	Interventions for each group with sufficient detail to allow replication, including how and when they will be administered NA
	11b	Criteria for discontinuing or modifying allocated interventions for a given trial participant (eg, drug dose change in response to harms, participant request, or improving/worsening disease) NA
	11c	Strategies to improve adherence to intervention protocols, and any procedures for monitoring adherence (eg, drug tablet return, laboratory tests) NA
	11d	Relevant concomitant care and interventions that are permitted or prohibited during the trial NA
Outcomes	12	Primary, secondary, and other outcomes, including the specific measurement variable (eg, systolic blood pressure), analysis metric (eg, change from baseline, final value, time to event), method of aggregation (eg, median, proportion), and time point for each outcome. Explanation of the clinical relevance of chosen efficacy and harm outcomes is strongly recommended Pages 6 – 9 under Intervention Description
Participant timeline	13	Time schedule of enrolment, interventions (including any run-ins and washouts), assessments, and visits for participants. A schematic diagram is highly recommended (see Figure) NA
Sample size	14	Estimated number of participants needed to achieve study objectives and how it was determined, including clinical and statistical assumptions supporting any sample size calculations Table 1 on page 9
Recruitment	15	Strategies for achieving adequate participant enrolment to reach target sample size NA

Methods: Assignment of interventions (for controlled trials)

Allocation:

Sequence generation	16a	Method of generating the allocation sequence (eg, computer-generated random numbers), and list of any factors for stratification. To reduce predictability of a random sequence, details of any planned restriction (eg, blocking) should be provided in a separate document that is unavailable to those who enrol participants or assign interventions NA

Allocation concealment mechanism	16b	Mechanism of implementing the allocation sequence (eg, central telephone; sequentially numbered, opaque, sealed envelopes), describing any steps to conceal the sequence until interventions are assigned NA
Implementation	16c	Who will generate the allocation sequence, who will enrol participants, and who will assign participants to interventions NA
Blinding (masking)	17a	Who will be blinded after assignment to interventions (eg, trial participants, care providers, outcome assessors, data analysts), and how NA
	17b	If blinded, circumstances under which unblinding is permissible, and procedure for revealing a participant's allocated intervention during the trial NA

Methods: Data collection, management, and analysis

Data collection methods	18a	Plans for assessment and collection of outcome, baseline, and other trial data, including any related processes to promote data quality (eg, duplicate measurements, training of assessors) and a description of study instruments (eg, questionnaires, laboratory tests) along with their reliability and validity, if known. Reference to where data collection forms can be found, if not in the protocol Table 2 on page 10
	18b	Plans to promote participant retention and complete follow-up, including list of any outcome data to be collected for participants who discontinue or deviate from intervention protocols NA
Data management	19	Plans for data entry, coding, security, and storage, including any related processes to promote data quality (eg, double data entry; range checks for data values). Reference to where details of data management procedures can be found, if not in the protocol NA
Statistical methods	20a	Statistical methods for analysing primary and secondary outcomes. Reference to where other details of the statistical analysis plan can be found, if not in the protocol NA
	20b	Methods for any additional analyses (eg, subgroup and adjusted analyses) NA
	20c	Definition of analysis population relating to protocol non-adherence (eg, as randomised analysis), and any statistical methods to handle missing data (eg, multiple imputation) NA

Methods: Monitoring

Data monitoring	21a	Composition of data monitoring committee (DMC); summary of its role and reporting structure; statement of whether it is independent from the sponsor and competing interests; and reference to where further details about its charter can be found, if not in the protocol. Alternatively, an explanation of why a DMC is not needed NA
	21b	Description of any interim analyses and stopping guidelines, including who will have access to these interim results and make the final decision to terminate the trial NA
Harms	22	Plans for collecting, assessing, reporting, and managing solicited and spontaneously reported adverse events and other unintended effects of trial interventions or trial conduct NA
Auditing	23	Frequency and procedures for auditing trial conduct, if any, and whether the process will be independent from investigators and the sponsor NA
**Ethics and dissemination**		
Research ethics approval	24	Plans for seeking research ethics committee/institutional review board (REC/IRB) approval On page 3 and page 11 under Ethics and dissemination
Protocol amendments	25	Plans for communicating important protocol modifications (eg, changes to eligibility criteria, outcomes, analyses) to relevant parties (eg, investigators, REC/IRBs, trial participants, trial registries, journals, regulators) NA
Consent or assent	26a	Who will obtain informed consent or assent from potential trial participants or authorised surrogates, and how (see Item 32) Page 9 under data collection
	26b	Additional consent provisions for collection and use of participant data and biological specimens in ancillary studies, if applicable NA
Confidentiality	27	How personal information about potential and enrolled participants will be collected, shared, and maintained in order to protect confidentiality before, during, and after the trial Page 13, under Ethics and Dissemination
Declaration of interests	28	Financial and other competing interests for principal investigators for the overall trial and each study site Page 17, under Competing interests statement
Access to data	29	Statement of who will have access to the final trial dataset, and disclosure of contractual agreements that limit such access for investigators Page 13, under Ethics and Dissemination
Ancillary and post-trial care	30	Provisions, if any, for ancillary and post-trial care, and for compensation to those who suffer harm from trial participation. NA

For peer review only

Dissemination policy	31a	Plans for investigators and sponsor to communicate trial results to participants, healthcare professionals, the public, and other relevant groups (eg, via publication, reporting in results databases, or other data sharing arrangements), including any publication restrictions. On page 12 under Ethics and dissemination
	31b	Authorship eligibility guidelines and any intended use of professional writers NA
	31c	Plans, if any, for granting public access to the full protocol, participant-level dataset, and statistical code The full study protocol has already been published and is mentioned in the reference list (no. 22). Accessibility to the process evaluation data has been detailed on page 12 under Ethics and dissemination
**Appendices**		
Informed consent materials	32	Model consent form and other related documentation given to participants and authorised surrogates PISCF shared as an attachment on the online portal .
Biological specimens	33	Plans for collection, laboratory evaluation, and storage of biological specimens for genetic or molecular analysis in the current trial and for future use in ancillary studies, if applicable NA

*It is strongly recommended that this checklist be read in conjunction with the SPIRIT 2013 Explanation & Elaboration for important clarification on the items. Amendments to the protocol should be tracked and dated. The SPIRIT checklist is copyrighted by the SPIRIT Group under the Creative Commons "Attribution-NonCommercial-NoDerivs 3.0 Unported" license.

For peer review only

BMJ Open

Protocol for process evaluation of ARTEMIS cluster randomized controlled trial: an intervention for management of depression and suicide among adolescents living in slums in India

Journal:	*BMJ Open*
Manuscript ID	bmjopen-2023-081844.R2
Article Type:	Protocol
Date Submitted by the Author:	12-Apr-2024
Complete List of Authors:	Mukherjee, Ankita; The George Institute for Global Health India,<br>Yatirajula, Sandhya Kanaka; The George Institute for Global Health India, Research<br>Kallakuri, Sudha; The George Institute for Global Health<br>Paslawar, Srilatha; The George Institute for Global Health India, Research and Development<br>Lempp, Heidi; King's College London, Inflammation Biology<br>Raman, Usha; University of Hyderabad, Hyderabad, India, Department of Communication,<br>Essue, Beverley; University of Toronto, Institute of Health Policy, Management and Evaluation<br>Sagar, Rajesh; All India Institute of Medical Sciences, Department of Psychiatry<br>Singh, Renu; Young Lives, India<br>Peiris, David; The George Institute for Global Health; University of New South Wales<br>Norton, Robyn; The George Institute for Global Health; Imperial College London<br>Thornicroft, Graham; Institute of Psychiatry, Psychology and Neuroscience, King's College London, Centre for Global Mental Health and Centre for Implementation Science<br>Maulik, Pallab; The George Institute for Global Health; University of New South Wales Medicine & Health
**Primary Subject Heading**:	Mental health
Secondary Subject Heading:	Mental health
Keywords:	Adolescents < Adolescent, Protocols & guidelines < HEALTH SERVICES ADMINISTRATION & MANAGEMENT, Implementation Science, MENTAL HEALTH, Clinical Trial, Depression & mood disorders < PSYCHIATRY

Protocol for process evaluation of ARTEMIS cluster randomized controlled trial: an intervention for management of depression and suicide among adolescents living in slums in India

Authors

1. Ankita Mukherjee (AM), The George Institute for Global Health, New Delhi, India, Email - AMukherjee@georgeinstitute.org.in
2. Sandhya Kanaka Yatirajula (SKY), Corresponding author), The George Institute for Global Health, New Delhi, India, Email – syatirajula@georgeinstitute.org.in
3. Sudha Kallakuri (SK), The George Institute for Global Health, Hyderabad, India, Email - skallakuri1@georgeinstitute.org.in
4. Srilatha Paslawar (SP), The George Institute for Global Health, Hyderabad, India, Email - SPaslawar@georgeinstitute.org.in
5. Heidi Lempp (HL), Centre for Rheumatic Diseases, Department of Inflammation Biology, School of Immunology and Microbial Sciences, Faculty of Life Sciences and Medicine, King's College London, UK, Email - heidi.lempp@kcl.ac.uk
6. Usha Raman (UR), Department of Communication, University of Hyderabad, Hyderabad, India, Email - usharaman@uohyd.ac.in
7. Beverley M. Essue (BE), Institute for Health Policy, Management and Evaluation, University of Toronto, Toronto, Canada, Email - beverley.essue@utoronto.ca
8. Rajesh Sagar (RS), Department of Psychiatry, All India Institute of Medical Sciences, New Delhi, Email - rsagar29@gmail.com
9. Renu Singh (ReS), Young Lives India, New Delhi, Email - renusab@gmail.com
10. David Peiris (DP), The George Institute for Global Health, UNSW Sydney, Sydney Australia, Email - dpeiris@georgeinstitute.org
11. Robyn Norton (RS), The George Institute for Global Health, UNSW Sydney, Sydney, Australia Imperial College, London, Email - rnorton@georgeinstitute.org
12. Graham Thornicroft (GT), Centre for Global Mental Health and Centre for Implementation Science, Institute of Psychiatry, Psychology and Neuroscience, King's College London, UK, Email - graham.thornicroft@kcl.ac.uk
13. Pallab K Maulik (PKM), The George Institute for Global Health, New Delhi, India, University of New South Wales, Sydney, Australia, Department of Brain Sciences, Imperial College London, London, United Kingdom, George Institute for Global Health, Imperial College London, United

For peer review only

Kingdom, Prasanna School of Public Health, Manipal Academy of Higher Education, Manipal, India, Email - pmaulik@georgeinstitute.org.in

For peer review only

Abstract

Introduction

There are around 250 million adolescents (10-19 years) in India. The prevalence of mental health related morbidity among adolescents in India is approximately 7.3%. Vulnerable sub-populations among adolescents such as those living in slum communities, are particularly at risk due to poor living conditions, financial difficulty, and limited access to support services The Adolescents' Resilience and Treatment nEeds for Mental Health in Indian Slums (ARTEMIS) is a cluster randomized controlled trial (cRCT) of an intervention intends to improve mental health of adolescents living in slum communities in India. The aim of this paper is to describe the process evaluation protocol for ARTEMIS trial. The process evaluation will help to explain the intervention outcomes and understand how and why intervention worked or did not work. It will identify contextual factors, intervention barriers and facilitators and the adaptations required for optimising implementation.

Methods

Case study method will be used and the data will include a mix of quantitative metrics and qualitative data. The UK Medical Research Council's guidance on evaluating complex interventions, the RE-AIM Framework and the APEASE criteria will be used to develop a conceptual framework and apriori codes for qualitative data analysis. Quantitative data will be analysed using descriptive statistics. Implementation fidelity will also be measured.

Discussion

The process evaluation will provide an understanding of outcomes and causal mechanisms that influenced any change in trial outcomes.

Ethics and Dissemination

Ethics Committee of the George Institute for Global Health India (Project number 17/2020) and the Research Governance and Integrity Team, Imperial College, London (ICREC Reference number: 22IC7718) have provided ethics approval. The Health Ministry's Screening Committee, has approved to the study (ID 2020-9770).

Trial Registration

The trial is registered with Clinical Trial Registry of India (Reference Number CTRI/2022/02/040307). The George Institute for Global Health, India is the trial sponsor.

Article Summary:

For peer review only

Strengths and limitations of this study

- This study will use existing implementation science theories and frameworks and qualitative and quantitative data will be triangulated to arrive at a comprehensive understanding of the intervention.
- This study will be guided by the MRC framework to developing and evaluating complex interventions and the case study method will also be used.
- Cases will be purposively selected based taking a maximum variation approach and both intervention and control sites will be selected as cases to the enable comparison, to understand contextual factors and to avoid the Hawthorne effect.

Key Words: process evaluation, protocol, Child and Adolescent Mental Health (CAMH), Low-middle income country (LMIC), cluster Randomized Control Trial (cRCT), depression, self-harm, anti-stigma, mHealth, electronic decision support system (EDSS), urban slum, India

Word count: 3459

For peer review only

INTRODUCTION

India has about 250 million adolescents aged 10-19 years, comprising nearly one-fifth of India's population [1]. The burden of mental health problems in adolescents is a growing concern globally[2]. In India, the prevalence of psychiatric disorders among adolescents is estimated to be about 7.3% [3]. Self-harm and depressive disorders are the leading cause of death and disability in the age group 15-19 years [4].

Mental health interventions for adolescents are important as 50% of adult mental disorders have an onset before age of 14 years[5]. Marginalized populations such as adolescents living in slum communities, are particularly vulnerable because of additional stressors related to poor living conditions, financial stress, and poor access to support services [6,7].

There has been limited research to test community-based mental health interventions for adolescents living in slums in India. A scoping review of mental health interventions among adolescents in India found that of the 11 interventions included in the review; nine were school-based, one community-based, and one was digital [8]). Most of the school-based programs used a life skills curriculum that resulted in improvements in depressive symptoms and overall mental well-being. The review recommended the need for more interventions for early and out-of-school adolescents to ensure that the most vulnerable adolescents were not missed out. An intervention to build mental health and resilience delivered by community-based peers among highly disadvantaged young women living in urban slums in Dehradun a city in north India, showed sustained improvements in anxiety and depression and attitudes to gender equality among study participants [9]. A randomized controlled trial of a 5-month resilience-based program among rural adolescent girls through government schools in the state of Bihar, India, delivered by local women showed that girls receiving the intervention (vs. controls) had better emotional resilience, self-efficacy, social-emotional assets, psychological wellbeing, and social wellbeing [10]. (Another school-based pilot study of POD (**P**roblems, **O**ptions, **D**o it!) Adventures (a smart phone based blended problem-solving game-based intervention for adolescents with or at risk of anxiety, depression and conduct difficulties) was helpful in managing their problems and stress and improving the mental health of 13 to 19 year olds enrolled in secondary schools in the Indian state of Goa [11]. The Adolescents' Resilience and Treatment nEeds for Mental Health in Indian Slums (ARTEMIS) cluster randomized control trial (cRCT) is testing a community-based intervention to improve mental health outcomes for adolescents living in urban slum clusters in India.

This paper presents the protocol for process evaluation of the ARTEMIS cRCT. ARTEMIS is a community-based cluster randomised control trial that aims to reduce depression and risk of suicide among adolescents living in slums. The intervention will use a mental health stigma reduction campaign with adolescents and parents of the study cohort to improve attitudes towards mental

For peer review only

health and improve help seeking. A technology-enabled strategy will be employed for screening, clinical diagnosis, and management of mental health problems (depression, other significant emotional or medically unexplained complaints and suicide risk) among adolescents by primary care doctors and community based non physician health workers (NPHWs) (described in detail below).

Process evaluations provide critical inputs in understanding how interventions work in particular contexts and thus, support implementation planning beyond the trial setting [12].

Aims

The aims of the process evaluation are to:

1. Assess implementation fidelity and understand how the intervention was implemented

2. Identify key contextual factors that impact intervention delivery and outcomes

3. Understand perceptions of different stakeholders about effectiveness, acceptability, and scalability of intervention components

4. Identify key facilitators and barriers in implementation of the intervention, and

5. Explain any adaptations to the intervention or intervention refinement during the study and their possible impact on the outcomes

METHODS

Conceptual Framework

The process evaluation will be guided by the MRC Framework for developing and evaluating complex interventions [13, 14]. The framework highlights the importance of examining the *implementation*, the *mechanism of impact* and their interaction with *contextual factors* to better understand how and why an intervention does or does not work. A recent update to the framework recognises that complex interventions have several phases including intervention development, feasibility assessment, implementation and evaluation, which may not always be sequential [15]. It recommends six core areas of inquiry at each phase before moving on to the next phase. They include (1) the intervention and its interaction with context; (2) the programme theory; (3) ways of engaging with diverse stakeholders; (4) ways of intervention refinement; (5) identifying key uncertainties; and (6) economic considerations. The process evaluation will focus on the first five areas of inquiry, and the overall objectives have been framed to address these key areas.

Study Setting

The ARTEMIS cRCT will be implemented in 60 slum clusters across two cities New Delhi and Vijayawada in India. In each city 30 slum clusters will be included. For this study, a slum cluster is defined as slums within wards or geographical areas identified as slums/resettlement colonies. A ward is a local authority area, typically used for electoral purposes. In certain cities of India, such as Mumbai and New Delhi, a ward is an administrative unit of the city region; a city area is divided into zones, which in turn contains numerous wards. New Delhi is a metropolis and one of the largest cities in India with a population of about 17 million and an estimated slum population of about 2 million [16,17]. Vijayawada is one of the largest cities in the state of Andhra Pradesh with an urban population of over 1.0 million[18,19] and an estimated slum population is estimated to be about 0.5 million[17]. The most widely spoken language in Delhi is Hindi, while in Vijayawada it is Telugu.

Patient and Public Involvement

Adolescent Expert Advisory Groups (AEAG) have been formed in each site. This group was involved throughout the intervention development phase in providing inputs and suggestions. Their contributions were critical for the co-creation of the anti-stigma content for adolescents. During the formative phase 34 meetings were held where the AEAG provided valuable feedback on ways to engage adolescents in the anti-stigma campaign.

Study Design

The study will use a mixed method, multiple case study design. Each slum cluster will constitute a 'case' for the study. A total of six cases or clusters will be purposively selected taking a maximum variation approach. Slums will be purposively selected to represent different contexts, coverage and reach of intervention as well as the ease or difficulty of implementing the intervention.

Intervention Description

The ARTEMIS cRCT will test an intervention to address depression, increased risk of self-harm/suicide, or other significant emotional or medically unexplained complaints among adolescents living in urban slums in two cities in India. ARTEMIS has two components. The first is a campaign that aims to reduce stigma related to mental health and improve attitudes and behaviours towards adolescents with depression or at increased risk of self-harm/suicide. The second is a technology-enabled mHealth platform with an integrated electronic decision support system (EDSS), to help primary care doctors and NPHWs to diagnose and treat adolescents at high-risk of depression, self-harm, or suicide.

Before randomization and before the start of the intervention, a team of trained field investigators will screen adolescents at high-risk of depression or suicide using Patient Health Questionnaire- 9 (PHQ-9), which is a standardised psychometric tool for screening depression and suicide risk in the

For peer review only

community [20, 21]. Adolescents who obtain a PHQ 9 score of ≥ 10 and/or a score of ≥2 to the suicide risk question on the PHQ-9 will be deemed as 'high-risk'. Due to the time delay between screening and randomisation and potential natural remission, a second screening will be carried out for adolescents identified at 'high-risk' before the baseline is administered. This process will help identify the final list of adolescents at 'high-risk' in all the clusters. The following information will be collected from the study cohort by the trained field investigators: socio-demographic characteristics, history of mental illness, treatment history, co-morbid conditions, stressful events experienced in the previous year, resilience, knowledge attitude and behaviours related to mental health and stigma associated with help seeking. A detailed protocol of the trial has been published [22].

A theory of change model for the intervention was developed with key stakeholders during the formative phase of the study in 2021. The logic model for intervention has been provided (Figure 1)

(Figure 1. Logic model about here)

Intervention Arm

In the intervention arm, the two components (i) the anti-stigma campaign and (ii) the technology-enabled mHealth based EDSS for screening, diagnosis and management of depression and self-harm will be delivered. (Figure 1).

Anti-stigma campaign

The anti-stigma campaign will include several Information Education and Communication (IEC) materials containing key messages around addressing mental health stigma, myths related to mental health, highlighting major stressors and their impact on mental health of adolescents and the importance of help seeking for mental health problems. The IEC materials will include posters, pamphlets, brochures, videos of persons with lived experience (PWLE), animation videos, audio dramas, games, magic shows, rallies, and street plays. The materials will be co-created with adolescents from the study sites. Adolescent Expert Advisory Groups (AEAGs) will be created in both locations consisting of adolescents from the community, with the purpose of involving them on working with the implementation team to co-create the anti-stigma campaign. Details of the process of forming AEAGs and their engagement in shaping the anti-stigma campaign has been published elsewhere [23].

Peer-educators

Based on findings from the formative phase of the study, peer groups were established comprising of adolescents from the community in the age group 13-19 years, some of whom were from the study

For peer review only

cohort. Their role will be to provide support to adolescents who approached them to discuss problems and to promote mental health awareness in the community. All the members of the peer groups will be provided basic training by the research team on mental health, safe practices while using social media, addiction and substance use and promoting mental wellbeing through engaging in pleasurable activities such a listening to music, sharing problems with trusted individuals, having friends, exercising, and eating healthily.

Technology-enabled EDSS

The EDSS platform has been developed to assist primary care doctors and NPHWs to screen, diagnose and manage depression, other significant emotional or medically unexplained complaints and suicide risk among adolescents. High-risk adolescents will be referred to the doctor electronically via the mHealth platform by the NPHWs. In each cluster, one doctor and several NPHWs will be trained. The NPHWs will be trained to use the EDSS to provide basic supportive advice to adolescents at high-risk of depression or self-harm, refer them to the primary care doctors, and track treatment advise given by the doctors and will follow-up for treatment adherence. The NPHWs will make regular home visits to encourage adolescents at high risk and their families to seek help from the doctor. The NPHWs will also use the EDSS to clinically monitor the severity of high-risk individuals at four months and at ten months after the start of the intervention. High-risk individuals will be provided referral cards to take to the primary care doctors. The primary care doctors will be trained to use an EDSS to clinically diagnose and manage adolescents with depression, other significant emotional or medically unexplained complaints and self-harm/suicide ideation based on WHO's mhGAP-Intervention Guide algorithm[24]. Adolescents needing specialist care will be referred to a psychiatrist in a government facility. The EDSS will also capture and store (on a cloud server) information regarding adolescents' visits and type of care provided by the primary care doctor-counselling/pharmacotherapy/combination of both/referral/follow-up. All data will be encrypted and protected using user-defined passwords. The information will be visible to the NPHWs, the project supervisors and research team only. A colour-coded traffic light system integrated in the application will assist the NPHWs to identify high-risk adolescents who need to be prioritised for followed up. The EDSS will also provide them with simple questions tailored to the priority level as indicated by the traffic-light coding system, that they can ask the adolescents during follow-up to ensure appropriate treatment adherence or follow-up with doctors or mental health specialists.

Control Arm

The control arm will receive enhanced usual care that will include general psychoeducation through pamphlets to raise awareness on common mental disorder in adolescents amongst the wider

For peer review only

community. Parents/guardians of adolescents identified at high-risk will be advised to consult a primary care doctor or mental health professional. Those with high scores on PHQ-9 (a score of >=15 on PHQ9) and/or at high risk of suicide (suicide score >=2), indicating severe depression and/or high risk of suicide will be asked to seek care immediately and will be given a list of health facilities (with contact details) where such treatment is available. All NPHWs will be asked to follow up with the adolescents and their families more intensively and will recommend that they visit a doctor/psychiatrist immediately.

Data Collection

Quantitative data for the process evaluation will draw on metrics collected sourced from the open-source medical record system (OpenMRS; (OpenMRS.org). The OpenMRS, is a standardized community-driven open-source software for storing and processing medical record information. Quantitative data will be collected throughout the intervention via the mHealth platform. This will include data on screening outcome, treatment seeking, follow ups, and treatment provided for the high-risk cohort. Data will also be used to understand usage patterns of the EDSS by primary care doctors and NPHWs. The mHealth platform will capture this data in tablets used by NPHWs and primary care doctors for screening, treatment, and follow-up of the high-risk cohort. This data will be utilised to assess reach, effectiveness, and service utilisation. Data on competency and fidelity measures will also be captured throughout the intervention.

Qualitative data collection will include key informant interviews (KII) and focus group discussions (FGDs) with various stakeholders including high-risk and non-high-risk cohort in both arms, parents, primary care doctors, NPHWs, members of the AEAG and peer leaders. A total of 33 FGDs (each with 6-12 participants) and 28 interviews are planned across the six purposively selected slum clusters equally divided between both sites. This will include four intervention clusters (two each in Delhi and Vijayawada) and 2 control clusters (one each in Delhi Vijayawada) (Table 1). The KIIs and FGDs with stakeholders will help capture information related to fidelity of the intervention and identify any gaps that could be addressed. Written informed consent will be sought from all participants selected for KIIs and FGDs.

(Table 1 about here)

Data Analysis

The conceptual framework for the study and how it integrates with the key parameters from the MRC frameworks is provided in Table 2.

(Table 2 about here)

For peer review only - http://bmjopen.bmj.com/site/about/guidelines.xhtml

The case study methodology will also be used as it is recognised to be suitable to study complex interventions in different contexts and helps to capture 'the complexity of the case, the relationship between the intervention and the context and how the intervention worked (or did not)' ([25]. Following Pfadenhauer et al (2016) we define context as 'a set of characteristics and circumstances that consist of active and unique factors that surround the implementation. As such it is not a backdrop for implementation but interacts, influences, modifies and facilitates or constrains the intervention and its implementation' [26]. We will be selecting both intervention and control sites as cases to avoid the Hawthorne effect. Two slum cluster in the control arm will also be selected for the case study to enable comparison, to understand contextual factors and any changes to usual care in the absence of an intervention. The importance of studying changes in control arms has been recognised as being useful in understanding intervention impact [27]. A multiple case study design will be use where each case will be analysed at the case level, but we will also pull together all the cases from the intervention and control arms for a more in-depth analysis and comparison. Basic descriptive analysis will be conducted with the quantitative data. Qualitative data will be transcribed by a professional vendor and the transcripts will be read by at least two members of the research team and coded. Coding will be carried out using a priori codes based on the conceptual framework (Table 3) with the help of N-Vivo 12 software.

(Table 3 about here)

Any additional codes emerging during the analysis will be added to the coding framework. Members of the research team will read transcripts separately and come up with codes. The research team will then compare and combine their codes to evaluate their fit and usefulness and will examine the differences in code to see if any new insights can be generated. The code list will be finalised after a discussion between the researchers to establish agreement among coders. Qualitative and quantitative data will be triangulated to arrive at a comprehensive understanding of the intervention. Triangulation helps in confirming the findings from quantitative and qualitative data thereby increasing validity as well as leading to a better understanding of phenomena being studied [28]

The quantitative data will be used understand implementation outcomes. Indicators to study implementation outcomes will be informed by the conceptual framework and relevant implementation science theories and frameworks (Table 2). These include the RE-AIM [29] (Reach, Efficacy, Adoption, Implementation and Maintenance) framework and the APEASE (Affordability, Practicability, Effectiveness and cost-effectiveness, Acceptability, Safety/Side Effects and , Equity) criteria for evaluation of behaviour change interventions [30-32]. The APEASE criteria has some similarities with RE-AIM, but includes two additional parameters, i.e., practicability and safety. An

For peer review only

intervention is practicable if 'it can be delivered as designed through the means intended to the target population'[30]. Data will be extracted from OpenMRS and descriptive statistics will be used to get totals, proportions and changes over time.

Assessment of Implementation Fidelity

Implementation fidelity refers to the extent to which an intervention was implemented as intended. We will assess fidelity in 3 components (a) delivery of anti-stigma campaign, (b) implementation of the mHealth component, including trainings. Indicators have been developed for all these components to measure frequency of exposure and coverage. The quality of training provided to primary doctors on the mhGAP, NPHWs on the priority listing app and peer educators on promoting mental wellbeing and competency will be assessed through various tools including a post training satisfaction survey, pre-post-test self-assessment checklists as well as though rating by trainers and staff on competency checklists.

Discussion

This paper describes the design of a mixed method process evaluation for the ARTEMIS cRCT, which involves an anti-stigma campaign and a mobile device-based decision support system for primary care doctors and non-physician health workers, to improve treatment of adolescents at high-risk of depression, other significant emotional or medically unexplained complaints and suicide risk.

The process evaluation will help us understand and explain key causal mechanisms that led to change. and will therefore strengthen the understanding of the implementation process by highlighting various barriers and facilitators. Furthermore, it will provide an understanding of how local context played a role in the way the intervention was implemented and help identify the need and impact of any adaptations made to the intervention. The process evaluation will provide stakeholder perspectives on aspects of the intervention that worked and those that need further adaptation.

Strengths and Limitations

The protocol was developed using existing implementation science theories and frameworks and combines qualitative and quantitative measures to understand key aspects of how the intervention was implemented and which aspects worked or need further improvement. The MRC framework, which is a comprehensive framework, widely used in the field of implementation science will be used. The case study method using maximum variation approach will be used to make suitable comparisons among different contexts and avoid Hawthorne. The ARTEMIS intervention does not directly address social determinants which impact mental health outcomes. In this study we will collect additional qualitative and quantitative data on social support, socioeconomic conditions, education, and the

For peer review only

local context of participants which will help to better understand and explain linkages between mental health outcomes and social determinants.

Ethics and Dissemination

The study has received formal ethics approval from the Ethics Committee of the George Institute for Global Health India on 04 September 2020 (Project number 17/2020). The study also received formal ethics approval from the Research Governance and Integrity Team, Imperial College, London on 8th June 2022 (ICREC Reference number: 22IC7718). The Health Ministry's Screening Committee (HMSC), Indian Council for Medical Research (ICMR) has also provided approval to the project (ID 2020-9770). Findings will be disseminated to study participants and other stakeholders at a policy symposium. All identifiable personal data will be stored in password protected secured servers located at The George Institute for Global, India office in Hyderabad. Only deidentified data will be disseminated. Data will be available with the PI on an accessible data repository, which can be accessed by other researchers, subject to a formal request to the PI to access the data for research purposes.

Trial Status

At the time of writing the paper, the intervention had started in both sites. Randomization was executed in Vijayawada and in Delhi on 12th December 2022. Intervention components were implemented in both the sites.

References

1. UNICEF: On My Mind: The State of the World's Children 2021. New York: United Nations Children's Fund; 2021. Available from: https://www.unicef.org/media/114636/file/SOWC-2021-full-report-English.pdf

2. Bor W, Dean AJ, Najman J, Hayatbakhsh R: Are child and adolescent mental health problems increasing in the 21st century? A systematic review. The Australian and New Zealand journal of psychiatry. 2014.48(7):606–16. Available from: https://pubmed.ncbi.nlm.nih.gov/24829198/

3. Gururaj G, Varghese M, Benegal V, Rao GN, Pathak K, Singh LK, Mehta RY, Ram D, Shibukumar TM, Kokane A, Lenin Singh RK, Chavan BS, Sharma P, Ramasubramanian C, Dalal PK, Saha PK , Deuri SP, Giri AK, Kavishvar AB, Sinha VK, Thavody J, Chatterji R, Akoijam MR: National Health Survey of India 2015-16: Prevalence, patterns and outcomes. Bangalore; 2016.

For peer review only

4. Joshi R, Alim M, Maulik PK, Norton R: A contemporary picture of the burden of death and disability in Indian adolescents: data from the Global Burden of Disease Study. International Journal of Epidemiology. 2017.46(6):2036. Available from: /pmc/articles/PMC5837726/

5. Patton GC, Sawyer SM, Santelli JS, Ross DA, Afifi R, Allen NB, Arora M, Azzopardi P, Baldwin W, Bonell C, Kakuma R, Kennedy E, Mahon J, McGovern T, Mokdad AH, Patel V, Petroni S, Reavley N, Taiwo K, Waldfogel J, Wickremarathne D, Barroso C, Bhutta Z, Fatusi AO, Mattoo A, Diers J, Fang J, Ferguson J, Ssewamala F, Viner RM: Our future: a Lancet commission on adolescent health and wellbeing. Lancet (London, England). 2016.387(10036):2423. Available from: /pmc/articles/PMC5832967/

6. Culbreth R, Swahn MH, Ndetei D, Ametewee L, Kasirye R: Suicidal Ideation among Youth Living in the Slums of Kampala, Uganda. International Journal of Environmental Research and Public Health 2018, Vol. 15, Page 298. 2018.15(2):298. Available from: https://www.mdpi.com/1660-4601/15/2/298/htm

7. Subbaraman R, Nolan L, Shitole T, Sawant K, Shitole S, Sood K, Nanarkar M, Ghannam J, Betancourt TS, Bloom DE, Patil-Deshmukh A: The psychological toll of slum living in Mumbai, India: a mixedmethods study. Social science & medicine (1982). 2014.119:155. Available from: /pmc/articles/PMC4252879/

8. Mehra, D., Lakiang, T., Kathuria, N., Kumar, M., Mehra, S., & Sharma, S. (2022). Mental Health Interventions among Adolescents in India: A Scoping Review. *Healthcare (Basel, Switzerland)*, *10*(2), 337. https://doi.org/10.3390/healthcare10020337

9. Mathias, K., Pandey, A., Armstrong, G. *et al.* Outcomes of a brief mental health and resilience pilot intervention for young women in an urban slum in Dehradun, North India: a quasi-experimental study. *Int J Ment Health Syst* **12**, 47 (2018). https://doi.org/10.1186/s13033-018-0226-y

10. Leventhal, Katherine Sachs et al. "Building psychosocial assets and wellbeing among adolescent girls: A randomized controlled trial." *Journal of adolescence* vol. 45 (2015): 284-95. doi:10.1016/j.adolescence.2015.09.011

11. Gonsalves, Pattie P et al. "App-based guided problem-solving intervention for adolescent mental health: a pilot cohort study in Indian schools." *Evidence-based mental health* vol. 24,1 (2021): 11-18. doi:10.1136/ebmental-2020-300194

For peer review only

12. Lockwood I., Walker RM, Latimer S., Chaboyer W., Cooke M., Gillespie BM. Process evaluations undertaken alongside randomised controlled trials in the hospital setting: A scoping review. Contemporary Clinical Trials Communications, Volume 26, 2022, 100894,ISSN 2451-8654, https://doi.org/10.1016/j.conctc.2022.100894

13. Moore GF, Audrey S, Barker M, Bond L, Bonell C, Hardeman W, Moore L, O'Cathain A, Tinati T, Wight D, Baird J: Process evaluation of complex interventions: Medical Research Council guidance. BMJ (Online). 2015.350.

14. Craig P, Dieppe P, Macintyre S, Mitchie S, Nazareth I, Petticrew M: Developing and evaluating complex interventions: The new Medical Research Council guidance. BMJ. British Medical Journal Publishing Group; 2008. p. 979–83. Available from: www.mrc.ac.uk/

15. Skivington K, Matthews L, Simpson SA, Craig P, Baird J, Blazeby JM, Boyd KA, Craig N, French DP, McIntosh E, Petticrew M, Rycroft-Malone J, White M, Moore L: A new framework for developing and evaluating complex interventions: update of Medical Research Council guidance. BMJ. 2021.374. Available from: https://www.bmj.com/content/374/bmj.n2061

16. Office of the Registrar General & Census Commissioner India: Primary Census Abstract Data Tables. [accessed 2022 Jun 29]. Available from: https://web.archive.org/web/20190714165104/http://censusindia.gov.in/pca/pcadata/pca.html

17. Ministry of Housing and Urban Poverty Alleviation Government of India: Slums in India : A Statistical Compendium 2015. 2015 [accessed 2022 Jul 19]. Available from: http://nbo.nic.in/pdf/SLUMS_IN_INDIA_Slum_Compendium_2015_English.pdf

18. Government of Andhra Pradesh: Handbook of Statistics 2019: Krishna District. 2019.

19. Office of the Registrar General & Census Commissioner: Population Finder 2011. Ministry of Home Affairs,Government of India [Internet]. Available from: https://censusindia.gov.in/census.website/data/population-finder

20. Pfizer Inc.: Patient Health Questionnaire- 9 (PHQ-9). 2010 [accessed 2020 Jul 20]. Available from: https://www.phqscreeners.com/images/sites/g/files/g10060481/f/201412/PHQ9_English for India.pdf

For peer review only

21. Kroenke K, Spitzer RL, Williams JBW: The PHQ-9: Validity of a brief depression severity measure. Journal of General Internal Medicine. 2001.16(9):606–13. Available from: /pmc/articles/PMC1495268/?report=abstract

22. Yatirajula SK, Kallakuri S, Paslawar S, Mukherjee A, Bhattacharya A, Chatterjee S, Sagar R, Kumar A, Lempp H, Raman U, Singh R, Essue B, Billot L, Peiris D, Norton R, Thornicroft G, Maulik PK: An intervention to reduce stigma and improve management of depression, risk of suicide/self-harm and other significant emotional or medically unexplained complaints among adolescents living in urban slums: protocol for the ARTEMIS project. Trials 2022 23:1. 2022.23(1):1–15. Available from: https://trialsjournal.biomedcentral.com/articles/10.1186/s13063-022-06539-8

23. Yatirajula SK, Kallakuri S, Paslawar S, Mukherjee A, Khan NA, Kumari K, Sagar R, Thornicroft G, Maulik PK: Engaging with adolescents to navigate the Adolescents' Resilience and Treatment nEeds for Mental health in Indian Slums (ARTEMIS) trial. Journal of Global Health.2022.12:03048

24. WHO: mhGAP Intervention Guide for mental, neurological and substance use disorders in non- specialized health settings (Version 1.0). Geneva; 2010 [accessed 2020 Jun 11]. Available from: www.who.int/mental_health/mhgap

25. Yin R. Case study research and applications: design and methods. Los Angeles: Sage Publications Inc; 2018

26. Pfadenhauer L, Rohwer A, Burns J, Booth A, Lysdahl KB, Hofmann B, Gerhardus A, Mozygemba K, Tummers M, Wahlster P, et al. Guidance for the assessment of context and implementation in health technology assessments (HTA) and systematic reviews of complex interventions: the Context and Implementation of Complex Interventions (CICI) framework: Integrate-HTA; 2016

27. Rachel Jewkes, Andrew Gibbs, Esnat Chirwa & Kristin Dunkle (2020) What can we learn from studying control arms of randomised VAW prevention intervention evaluations: reflections on expected measurement error, meaningful change and the utility of RCTs, Global Health Action, 13:1, DOI: 10.1080/16549716.2020.1748401

28. Bekhet Ak., Zauszniewski JA. Methodological triangulation: an approach to understanding data. Nurse Researcher. 20, 2, 40-43. doi: 10.7748/nr2012.11.20.2.40.c9442

For peer review only

29. Waitzkin H: Different Views A Marxian Interpretation of the Growth And Development of Coronary Care Technology. American Journal of Public Health. 1979.69(12):1260–8. Available from: http://ajph.aphapublications.org/doi/pdf/10.2105/AJPH.69.12.1260

30. Michie S, Atkins L, Gainforth HL: Changing Behaviour to Improve Clinical Practice and Policy. In: Gonçalves A, Azevedo A, Lobo F, editors. Novos Desafios, Novas Competências: Contributos Atuais da Psicologia. Braga,Portugal: Axioma - Publicações da Faculdade de Filosofia; 2016. p. 41–60.

31. Michie S, West R, Rogers MB, Bonell C, Rubin GJ, Amlôt R: Reducing SARS-CoV-2 transmission in the UK: A behavioural science approach to identifying options for increasing adherence to social distancing and shielding vulnerable people. British Journal of Health Psychology. 2020.25(4):945–56.

32. Brierley ML, Smith LR, Bailey DP, Ojo SO, Hewson DJ, Every SA, Staines TA, Chater AM: Evaluating a multi-component intervention to reduce and break up office workers' sitting with sit-stand desks using the APEASE criteria. BMC Public Health. 2022.22(1):1–15. Available from: https://bmcpublichealth.biomedcentral.com/articles/10.1186/s12889-022-12794-w

Author contribution statement

The paper was conceptualised by PKM. AM wrote the original draft with contributions from SKY. PKM commented on multiple drafts before sending a prefinal version to everyone listed as authors. SK, SP, HL, UR, BME, RS, ReS, DP, RN, GT reviewed the draft and provided critical intellectual inputs and comments to the draft. PKM leads the implementation of the trial in India along with SKY, SK, SP and AM. SKY, SK and SP played a key role in implementing research activities in the sites. All authors read and approved the final manuscript.

Funding

Adolescents' Resilience and Treatment nEeds for Mental health in Indian Slums (ARTEMIS) is funded by UK Research and Innovation/Medical Research Council (UKRI/MRC), Grant no: MR/S023224/1. SKY, SK, and SP are all partially or fully supported by ARTEMIS. PKM and AM are partially supported through NHMRC/GACD grant (SMART MentalHealth-APP1143911) and ARTEMIS. PKM is the PI for ARTEMIS and Co-PI for SMART Mental Health. HL and GT are partly support by ARTEMIS. GT is also supported by the National Institute for Health Research (NIHR) Applied Research Collaboration South London at King's College London. The views expressed are those of the author(s) and not necessarily those of the NHS, the NIHR or the Department of Health and Social Care. GT is also supported by the MRC-UKRI in

For peer review only

relation to the Indigo Partnership (MR/R023697/1) awards. The funding agency had no role in the writing of the manuscript or the decision to submit it for publication.

Competing interests statement

We declare that the George Institute for Global Health has a part-owned social enterprise, George Health Enterprises, which has commercial relationships involving digital health innovations.

Acknowledgements

We acknowledge the contribution of all members of the Adolescent Expert Advisory Group in Delhi and Vijayawada who has generously contributed their time and expertise to the development of ARTEMIS. GT is supported by the National Institute for Health and Care Research (NIHR) Applied Research Collaboration South London (NIHR ARC South London) at King's College Hospital NHS Foundation Trust. The views expressed are those of the author(s) and not necessarily those of the NIHR or the Department of Health and Social Care.

Data Availability Statement

This is a process evaluation protocol paper; no data has been collected as yet and therefore there is no data that can be shared. However, the data will be made available on reasonable request after publication of findings.

For peer review only

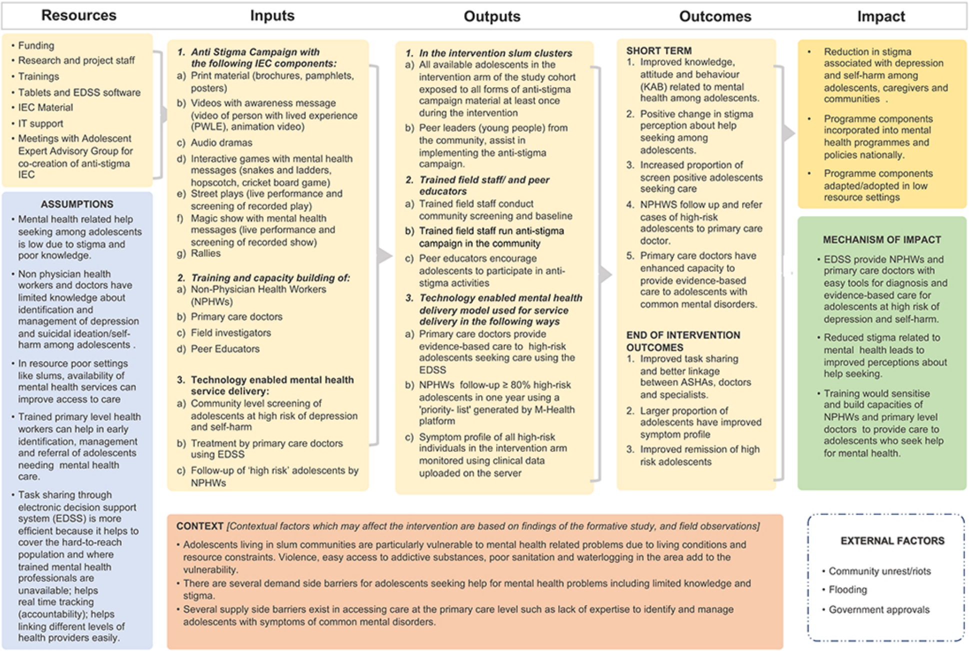

90x60mm (300 x 300 DPI)

Table 1: Qualitative Data Collection Plan

**Intervention Arm**		
Data Sources	**FGDs Planned**	**Interviews planned**
Non-physician Health Workers (NPHWs)	4	-
Adolescents from high-risk cohort	8	-
Adolescents from nonhigh-risk cohort	4	
Parents of high-risk adolescents in the study cohort		24
Members of Adolescent Expert Advisory Groups (AEAG)	5	-
Peer Leaders	4	-
Field Staff	2	-
Key implementation team members	1	
Primary Care Doctors	-	4
**Control Arm**		
Non-Physician Health Workers (NPHWs)	2	-
Adolescents from study cohort	4	-
TOTAL	34	28

For peer review only

Table 2. Conceptual Framework for Process Evaluation

Broad Area of Enquiry	Domains of Inquiry	Key Questions/Process Measures	Data Source
CONTEXT	Differences in context	• What are the differences in terms of geography, slum conditions, socio-cultural norms, economic between the sites and among the clusters?<br>• What are the contextual factors which affect programme delivery, stakeholder response and intervention uptake in different settings?<br>• Who were the key stakeholders who could play a role in improving intervention outcomes?	Secondary data;<br>Formative research data<br>FGDs with project staff<br>FGDs with key members of the implementation team
	Intervention adaptions based on context	• What are some of the key contextual factors which influenced the overall implementation of the intervention?<br>• What were some of the context specific adaptations that were made to address emerging challenges?	FGDs with project staff<br>FGDs with key members of the implementation team<br>Project documentation completed during the project
	Contextual Barriers and Facilitators	• What are key facilitators in different contexts that helped in intervention delivery and uptake?	FGDs with project staff

For peer review only

		• What are the barriers in various contexts for implementing the intervention components?	FGDs with key members of the implementation team

**IMPLEMENTATION**	Implementation fidelity	Was the intervention delivered as it was planned?	Program records and documents; Observation and rating
	Intervention Reach	• What was the coverage of the different *anti-stigma campaign* methods, in terms of:<br>- Total persons reached (including age and gender-wise break-up)<br>- Slums and clusters covered.<br>- Number and proportion of high-risk cohort reached.<br>- Number and proportion of non-high-risk cohort reached.<br>- Key stakeholders reached.<br>• What was the reach of the mHealth services:<br>- Number and proportion of high-risk cohort in the intervention arm provided counselling or follow-up services by NPHWs.	Project records and documents<br><br>Backend data<br><br>FGDs with project staff<br><br>FGDs with key members of the implementation team<br><br>Interviews with NPHWs

For peer review only

		- Number and proportion of high-risk cohort from the intervention arm who sought care with the primary care doctor.<br>- Number and proportion high risk-cohort from the control arm who sought care.<br><br>• Did the NPHWs face any challenge in reaching out adolescents and their families during the intervention?	
	Intervention effectiveness	• What was the perception of adolescents and key stakeholders about the utility effectiveness of the anti-stigma campaign?<br>• What are some of the key take home messages from anti stigma campaign for adolescents and community members?<br>• What was the perception of NPHWs about impact of anti-stigma campaign in their community?<br>• What is the association between exposure to anti stigma content with changes in KAB scores and care seeking?	Community satisfaction survey done at the end of street play performance.<br><br>Outcome survey data;<br>Backend data<br><br>FGDs with adolescents<br><br>FGDs with AEAG and peer leaders<br><br>Interviews with parents

For peer review only

		• What is the perception of NPHWs about effectiveness of mHealth based EDSS for management of depression and self-harm in adolescents?<br>• What is the perception of doctors about effectiveness of mHealth based EDSS for community-based management of depression and self-harm in adolescents?	
	Intervention acceptability and adoption	• What was the experiences of NPHWs in using EDSS for providing care (challenges, perceived benefits, potential for routine use of mHealth)?<br>• What was the experiences of doctors about using EDSS for providing care (challenges, perceived benefits, potential for routine use of mHealth)?<br><br>• What were some key features of use of EDSS by doctors:<br>- Average time taken for diagnosis and identification of treatment plan using mhGAP over time.<br>- Association between type of CMD and time taken for diagnosis and identification of treatment plan	Back-end data<br><br>FGDs with NPHWs<br>Interviews with doctors<br><br>Interview with high-risk adolescents and their parents

For peer review only

		using mhGAP.<br><br>• What were the perceptions of high-risk adolescents and their parents about ease of getting treatment through mHealth?	
	Practicability	• What were the challenges/barriers in delivering of the different strategies of anti-stigma campaign to adolescents?<br>• What were the challenges of the electronic decision support systems by NPHWs and doctors for diagnosis and management of depression and self-harm among adolescents?<br>• Doctor's experiences of interactions with adolescents<br>• Experiences of NPHWs with adolescents during follow up and motivating them and their families to visit the doctor initially and for follow up	FGDs with NPHWs<br><br>Interviews with doctors<br><br>FGDs with project staff
	Safety and Side Effects	• Were there any unanticipated negative consequences because of the anti-stigma campaign?	FGDs with adolescents<br><br>FGDs with NPHWs<br><br>Interviews with doctors

For peer review only

		• Were there any safety concerns related to using mHealth based EDSS platform for management of depression and self-harm among adolescents?	FGDs with project staff

**MECHANISM OF IMPACT**	Variation in outcomes	What kind of cluster level variation is observed in in the outcomes? What works, for whom and in what context?	Outcome data<br>Backend data;<br>FGDs with NPHWs<br>Interviews with doctors<br>FGDs with project staff
	Unexpected outcomes	What are some unexpected outcomes and what factors can be attributed to them?	Outcome data<br>Back-end data;<br>FGDs with NPHWs<br>Interviews with doctors<br>FGDs with project staff

For peer review only

	Theory of Change (ToC)/ Pathways to achieving outcomes	What were some of the key pathways to improved outcomes for high-risk adolescents?<br>What changes and adaptations were made to the ToC during the intervention?<br>What were some of the key assumptions regarding causal mechanisms that need to be modified?	Outcome data<br>Back-end data<br>Project documents<br>FGDs with project staff

For peer review only

Table 3: Qualitative Data Collection Areas of Inquiry

Type of Group/Individual	Some areas of inquiry
Non-Physician Health Workers	• Perceptions on effectiveness and appropriateness of training received.<br>• Experience of using mHealth platform for delivering the intervention including challenges or facilitators<br>• Perception about effectiveness anti-stigma campaign in improving help seeking.<br>• Facilitators and barriers in treatment seeking by high-risk cohort.<br>• Overall experience of participating in the trial.<br>• Perception about any personal benefits/harms due to their association with the project.<br>• Perception about what role they see for themselves in the community once the project comes to an end.<br>• Any spillover effects-experiences of helping or sharing information with other adolescents not in the study cohort.
Project Field staff in each site	• Barriers or facilitators in delivering anti-stigma campaign in the community.<br>• Barriers and facilitators in delivering mHealth component.<br>• Perceived factors which explain high/low treatment seeking in different clusters.<br>• Key lessons learnt and suggestions for implementation of intervention components.<br>• Perceptions on training appropriateness, effectiveness and methods.
Study participants from high-risk cohort in intervention arm	• Perception appropriateness of anti-stigma content and methods for adolescents.<br>• Perceptions about impact and effectiveness of anti-stigma campaign in community.<br>• Any reported change in perception about mental health related stigma and help seeking in self or family.

For peer review only

	• Perception about case detection, treatment and follow-up through EDSS.<br>• Facilitators and barriers in help-seeking by adolescents<br>• Experience of care and perception about quality of care, provided by doctors.<br>• Positive/negative experiences as a study participant.<br>• Perception about benefits/effectiveness of the intervention.<br>• Any spillover effects-experience of helping or sharing information with other adolescents not in the study cohort.
Study participants from non-high-risk cohort in intervention arm	• Perceptions about impact and effectiveness of anti-stigma campaign in community.<br>• Any reported changes in perception about mental health related stigma and help seeking in self or family.<br>• Facilitators and barriers in treatment seeking.<br>• Positive/negative experiences as a study participant.<br>• Perception about benefits/effectiveness of the intervention.<br>• Any spillover effects-experience of helping or sharing information with other adolescents not in the study cohort.
Study participants from (high-risk and non-high-risk cohort) in the control arm	• Perception about impact and effectiveness of anti-stigma content.<br>• Any reported change in perception about mental health related stigma and help seeking in self or family.<br>• Facilitators and barriers in help seeking.<br>• Experience as a study participant.
Parents of adolescents in the study cohort	• Perception about impact and effectiveness of anti-stigma content.<br>• Any reported change in perception about mental health related stigma and help seeking in self or family.

For peer review only

	• Knowledge about key stressors and symptoms of mental health problems in adolescents.<br>• Facilitators and barriers in help-seeking by adolescents.<br>• Experience of help seeking.<br>• Perception about benefits/effectiveness of the intervention.
Adolescent expert Advisory Groups in each site	• Perception about their role on co-creation of anti-stigma material and feedback on the whether the anti-stigma material was able reflect their suggestions.<br>• Perception about impact and effectiveness of anti-stigma content among adolescents and community members.<br>• Any reported change in perception about mental health related stigma and help seeking in self or family.<br>• Perceived personal benefit/challenges in being an AEAG member.<br>• Perception about benefit and effectiveness of mHealth component.<br>• Facilitators and barriers in help seeking by high-risk adolescents.<br>• Suggestions on improving engagement/involvement of younger adolescents in AEAGs and the co-creation process.
Peer Group -Intervention arm	• Perceptions on effectiveness and appropriateness of training received.<br>• Perceptions about impact and effectiveness of anti-stigma campaign in community.<br>• Perception about effectiveness anti-stigma campaign in improving help seeking by adolescents.<br>• Facilitators and barriers in treatment seeking by high-risk cohort.<br>• Experience if any of providing help to adolescents in the community who reached out to the peer group.<br>• Overall experiences of participating in the trial.<br>• How does the learnings from the project/trainings will be useful beyond the project period.

For peer review only

Doctors from intervention arm	• Perceptions on effectiveness and appropriateness of training received.<br>• Experience of using technology-based decision support system for diagnosis and management of depression and self-harm among adolescents.<br>• Perceived effectiveness anti-stigma campaign.<br>• Possible facilitators and barriers to scaling up mHealth component.<br>• Overall experience of participating in the trial including any challenges.<br>• Facilitators and barriers in help seeking and treatment adherence among adolescents.<br>• Whether learnings from the project/trainings will be useful beyond the project period.
Study participants from high-risk cohort in intervention arm who visited the doctor and were given medical and/or psychological treatment.	• Experience of seeking care from primary care doctor and perception about quality of care.<br>• Level of comfort/discomfort in sharing their mental distress experiences with doctors.<br>• Perceived benefit, if any, as a result of treatment.<br>• Barriers and facilitators for seeking treatment from a specialist (in case of referral).<br>• Views about help-seeking in future or continuing care if advised by the doctor.<br>• Positive/negative experiences as a study participant.<br>• Suggestions for the programme in the future.
Parents/guardians of study participants from high-risk cohort in intervention arm who visited the doctor	• Perception about impact and effectiveness of anti-stigma content.<br>• Any reported change in perception about mental health related stigma and help seeking in self or family.<br>• Knowledge about key stressors and symptoms of mental health problems in adolescents.<br>• Knowledge perception about mental health problems of their ward.<br>• Barriers and facilitators for seeking mental healthcare.

For peer review only

	• Experience of seeking mental health care for their wards.<br>• Perceived benefit, if any, as a result of treatment.<br>• Views about help-seeking in future or continuing mental health care if advised by the doctor.<br>• Suggestions for the programme in the future.

For peer review only

SPIRIT

STANDARD PROTOCOL ITEMS: RECOMMENDATIONS FOR INTERVENTIONAL TRIALS

SPIRIT 2013 Checklist: Recommended items to address in a clinical trial protocol and related documents*

Section/item	Item No	Description
**Administrative information**		
Title	1	(Page 1)
Trial registration	2a	(Page 3)
	2b	NA. Clinical Trials Registry of India is a listed registry under WHO
Protocol version	3	
Funding	4	Funding Statement on page 16
Roles and responsibilities	5a	Authors Contribution on page 15
	5b	Page 3 under Trial registration section
	5c	Funding Statement on page 16
	5d	This is a process evaluation protocol so these do not apply in general but the whole evaluation will be overseen by the research team led by the PI
**Introduction**		
Background and rationale	6a	Description of research question and justification for undertaking the trial, including summary of relevant studies (published and unpublished) examining benefits and harms for each intervention Page 4-5
	6b	Explanation for choice of comparators Page 10
Objectives	7	Specific objectives or hypotheses Page 5 under Aims
Trial design	8	Description of trial design including type of trial (eg, parallel group, crossover, factorial, single group), allocation ratio, and framework (eg, superiority, equivalence, noninferiority, exploratory) Page 6 under study design
**Methods: Participants, interventions, and outcomes**		
Study setting	9	Description of study settings (eg, community clinic, academic hospital) and list of countries where data will be collected. Reference to where list of study sites can be obtained Page 6 under Study Setting

For peer review only

Eligibility criteria	10	Inclusion and exclusion criteria for participants. If applicable, eligibility criteria for study centres and individuals who will perform the interventions (eg, surgeons, psychotherapists) NA
Interventions	11a	Interventions for each group with sufficient detail to allow replication, including how and when they will be administered NA
	11b	Criteria for discontinuing or modifying allocated interventions for a given trial participant (eg, drug dose change in response to harms, participant request, or improving/worsening disease) NA
	11c	Strategies to improve adherence to intervention protocols, and any procedures for monitoring adherence (eg, drug tablet return, laboratory tests) NA
	11d	Relevant concomitant care and interventions that are permitted or prohibited during the trial NA
Outcomes	12	Primary, secondary, and other outcomes, including the specific measurement variable (eg, systolic blood pressure), analysis metric (eg, change from baseline, final value, time to event), method of aggregation (eg, median, proportion), and time point for each outcome. Explanation of the clinical relevance of chosen efficacy and harm outcomes is strongly recommended Pages 6 – 9 under Intervention Description
Participant timeline	13	Time schedule of enrolment, interventions (including any run-ins and washouts), assessments, and visits for participants. A schematic diagram is highly recommended (see Figure) NA
Sample size	14	Estimated number of participants needed to achieve study objectives and how it was determined, including clinical and statistical assumptions supporting any sample size calculations Table 1 on page 9
Recruitment	15	Strategies for achieving adequate participant enrolment to reach target sample size NA

Methods: Assignment of interventions (for controlled trials)

Allocation:

Sequence generation	16a	Method of generating the allocation sequence (eg, computer-generated random numbers), and list of any factors for stratification. To reduce predictability of a random sequence, details of any planned restriction (eg, blocking) should be provided in a separate document that is unavailable to those who enrol participants or assign interventions NA

Allocation concealment mechanism	16b	Mechanism of implementing the allocation sequence (eg, central telephone; sequentially numbered, opaque, sealed envelopes), describing any steps to conceal the sequence until interventions are assigned NA
Implementation	16c	Who will generate the allocation sequence, who will enrol participants, and who will assign participants to interventions NA
Blinding (masking)	17a	Who will be blinded after assignment to interventions (eg, trial participants, care providers, outcome assessors, data analysts), and how NA
	17b	If blinded, circumstances under which unblinding is permissible, and procedure for revealing a participant's allocated intervention during the trial NA

Methods: Data collection, management, and analysis

Data collection methods	18a	Plans for assessment and collection of outcome, baseline, and other trial data, including any related processes to promote data quality (eg, duplicate measurements, training of assessors) and a description of study instruments (eg, questionnaires, laboratory tests) along with their reliability and validity, if known. Reference to where data collection forms can be found, if not in the protocol Table 2 on page 10
	18b	Plans to promote participant retention and complete follow-up, including list of any outcome data to be collected for participants who discontinue or deviate from intervention protocols NA
Data management	19	Plans for data entry, coding, security, and storage, including any related processes to promote data quality (eg, double data entry; range checks for data values). Reference to where details of data management procedures can be found, if not in the protocol NA
Statistical methods	20a	Statistical methods for analysing primary and secondary outcomes. Reference to where other details of the statistical analysis plan can be found, if not in the protocol NA
	20b	Methods for any additional analyses (eg, subgroup and adjusted analyses) NA
	20c	Definition of analysis population relating to protocol non-adherence (eg, as randomised analysis), and any statistical methods to handle missing data (eg, multiple imputation) NA

Methods: Monitoring

Data monitoring	21a	Composition of data monitoring committee (DMC); summary of its role and reporting structure; statement of whether it is independent from the sponsor and competing interests; and reference to where further details about its charter can be found, if not in the protocol. Alternatively, an explanation of why a DMC is not needed NA
	21b	Description of any interim analyses and stopping guidelines, including who will have access to these interim results and make the final decision to terminate the trial NA
Harms	22	Plans for collecting, assessing, reporting, and managing solicited and spontaneously reported adverse events and other unintended effects of trial interventions or trial conduct NA
Auditing	23	Frequency and procedures for auditing trial conduct, if any, and whether the process will be independent from investigators and the sponsor NA
**Ethics and dissemination**		
Research ethics approval	24	Plans for seeking research ethics committee/institutional review board (REC/IRB) approval On page 3 and page 11 under Ethics and dissemination
Protocol amendments	25	Plans for communicating important protocol modifications (eg, changes to eligibility criteria, outcomes, analyses) to relevant parties (eg, investigators, REC/IRBs, trial participants, trial registries, journals, regulators) NA
Consent or assent	26a	Who will obtain informed consent or assent from potential trial participants or authorised surrogates, and how (see Item 32) Page 9 under data collection
	26b	Additional consent provisions for collection and use of participant data and biological specimens in ancillary studies, if applicable NA
Confidentiality	27	How personal information about potential and enrolled participants will be collected, shared, and maintained in order to protect confidentiality before, during, and after the trial Page 13, under Ethics and Dissemination
Declaration of interests	28	Financial and other competing interests for principal investigators for the overall trial and each study site Page 17, under Competing interests statement
Access to data	29	Statement of who will have access to the final trial dataset, and disclosure of contractual agreements that limit such access for investigators Page 13, under Ethics and Dissemination
Ancillary and post-trial care	30	Provisions, if any, for ancillary and post-trial care, and for compensation to those who suffer harm from trial participation. NA

For peer review only

Dissemination policy	31a	Plans for investigators and sponsor to communicate trial results to participants, healthcare professionals, the public, and other relevant groups (eg, via publication, reporting in results databases, or other data sharing arrangements), including any publication restrictions. On page 12 under Ethics and dissemination
	31b	Authorship eligibility guidelines and any intended use of professional writers NA
	31c	Plans, if any, for granting public access to the full protocol, participant-level dataset, and statistical code The full study protocol has already been published and is mentioned in the reference list (no. 22). Accessibility to the process evaluation data has been detailed on page 12 under Ethics and dissemination
**Appendices**		
Informed consent materials	32	Model consent form and other related documentation given to participants and authorised surrogates PISCF shared as an attachment on the online portal .
Biological specimens	33	Plans for collection, laboratory evaluation, and storage of biological specimens for genetic or molecular analysis in the current trial and for future use in ancillary studies, if applicable NA

*It is strongly recommended that this checklist be read in conjunction with the SPIRIT 2013 Explanation & Elaboration for important clarification on the items. Amendments to the protocol should be tracked and dated. The SPIRIT checklist is copyrighted by the SPIRIT Group under the Creative Commons "Attribution-NonCommercial-NoDerivs 3.0 Unported" license.

For peer review only
